# Identification and validation of CCL2 as a potential biomarker relevant to mast cell infiltration in the testicular immune microenvironment of spermatogenic dysfunction

**DOI:** 10.1186/s13578-023-01034-2

**Published:** 2023-05-23

**Authors:** Fan Dong, Ping Ping, Si-Qi Wang, Yi Ma, Xiang-Feng Chen

**Affiliations:** 1 Center for Reproductive Medicine, School of Medicine, Ren Ji Hospital, Shanghai Jiao Tong University, 845 Lingshan Road, Shanghai, 200135 People’s Republic of China; 2 Shanghai Key Laboratory for Assisted Reproduction and Reproductive Genetics, Shanghai, China; 3 Shanghai Human Sperm Bank, Shanghai, China

**Keywords:** Spermatogenic dysfunction, CCL2, Testicular mast cells, Azoospermia, Testicular immune microenvironment

## Abstract

**Background:**

Spermatogenic dysfunction is an important cause of azoospermia. Numerous studies have focused on germ-cell-related genes that lead to spermatogenic impairment. However, based on the immune-privileged characteristics of the testis, the relationship of immune genes, immune cells or immune microenvironment with spermatogenic dysfunction has rarely been reported.

**Results:**

Using integrated methods including single-cell RNA-seq, microarray data, clinical data analyses and histological/pathological staining, we found that testicular mast cell infiltration levels were significantly negatively related to spermatogenic function. We next identified a functional testicular immune biomarker, CCL2, and externally validated that testicular CCL2 was significantly upregulated in spermatogenic dysfunctional testes and was negatively correlated with Johnsen scores (JS) and testicular volumes. We also demonstrated that CCL2 levels showed a significant positive correlation with testicular mast cell infiltration levels. Moreover, we showed myoid cells and Leydig cells were two of the important sources of testicular CCL2 in spermatogenic dysfunction. Mechanistically, we drew a potential “myoid/Leydig cells-CCL2-ACKR1-endothelial cells-SELE-CD44-mast cells” network of somatic cell–cell communications in the testicular microenvironment, which might play roles in spermatogenic dysfunction.

**Conclusions:**

The present study revealed CCL2-relevant changes in the testicular immune microenvironment in spermatogenic dysfunction, providing new evidence for the role of immunological factors in azoospermia.

**Supplementary Information:**

The online version contains supplementary material available at 10.1186/s13578-023-01034-2.

## Background

Infertility occurs in up to 12% of all male population [1]. The etiology of male infertility varies, but spermatogenic dysfunction, featured by the existence of defects in spermatogenesis function of testes, is the most common component in male infertility [2]. Although semen analysis roughly assesses the occurrence of spermatogenic dysfunction, its final diagnosis and grading assessment rely on pathological evaluation, especially for non-obstructive azoospermia (NOA) patients. Johnsen introduced a 10-point scoring system to evaluate testicular spermatogenic functions [3], known as the Johnsen score (JS), which has been modified by other researchers [4, 5] (modified JS, mJS), and has become a widely used pathological scoring system evaluating testicular spermatogenesis. Although the JS/mJS (hereafter similarly abbreviated as JS) precisely evaluates the severity of spermatogenic dysfunction, it fails to reveal the detailed mechanism of what happened in the dysfunctional testis. Therefore, revealing the mechanisms of spermatogenic dysfunction and identifying reliable molecular biomarkers are crucial for future research.

The testis is an immune privileged organ [6]. Adjacent Sertoli cells use tight junctions to form the blood-testis barrier (BTB), which protects spermatogenic process from the harm of immune system [7]. However, numerous studies have revealed that different sorts of immune cells also exist in the testicular microenvironment [8]. The infiltration level of mast cells has been shown to be significantly increased in spermatogenic dysfunctional testes compared to testes with normal spermatogenesis [9–11], indicating that mast cells may be involved in the development of spermatogenic dysfunction. However, these previous researches failed to reveal the detailed relationship between mast cell infiltration and spermatogenic dysfunction. Therefore, it remains unknown whether there is a biomarker related to both spermatogenic function and testicular mast cell infiltration, and it is unclear how testicular mast cells interact with other types of testicular cells.

Single-cell RNA sequencing (scRNA-seq) has been used to investigate different cell types in the testicular microenvironment. However, most of the previous scRNA-seq studies have focused on germ cells or non-immune somatic cells [12–17], and none of these studies discussed testicular mast cells or mast cell-related biomarkers in spermatogenic dysfunction. In the present study, through integrated analyses of scRNA-seq data, microarray data and paraffin-embedded samples, we explored the changes in the testicular immune microenvironment to screen and validate the biomarker that related to both testicular mast cell infiltration and spermatogenic dysfunction. Besides, we mapped the biomarker-mediated cell–cell communication networks and detected the biological pathways underlying spermatogenic dysfunction. We hope this study could provide a better understanding of the role of the testicular immune microenvironment to help identify therapeutic targets for spermatogenic dysfunction, especially for NOA.

## Results

### Landscape of testicular infiltrating immune cells and confirmation of the relationship between testicular mast cells and spermatogenic function

**Fig. 1:**
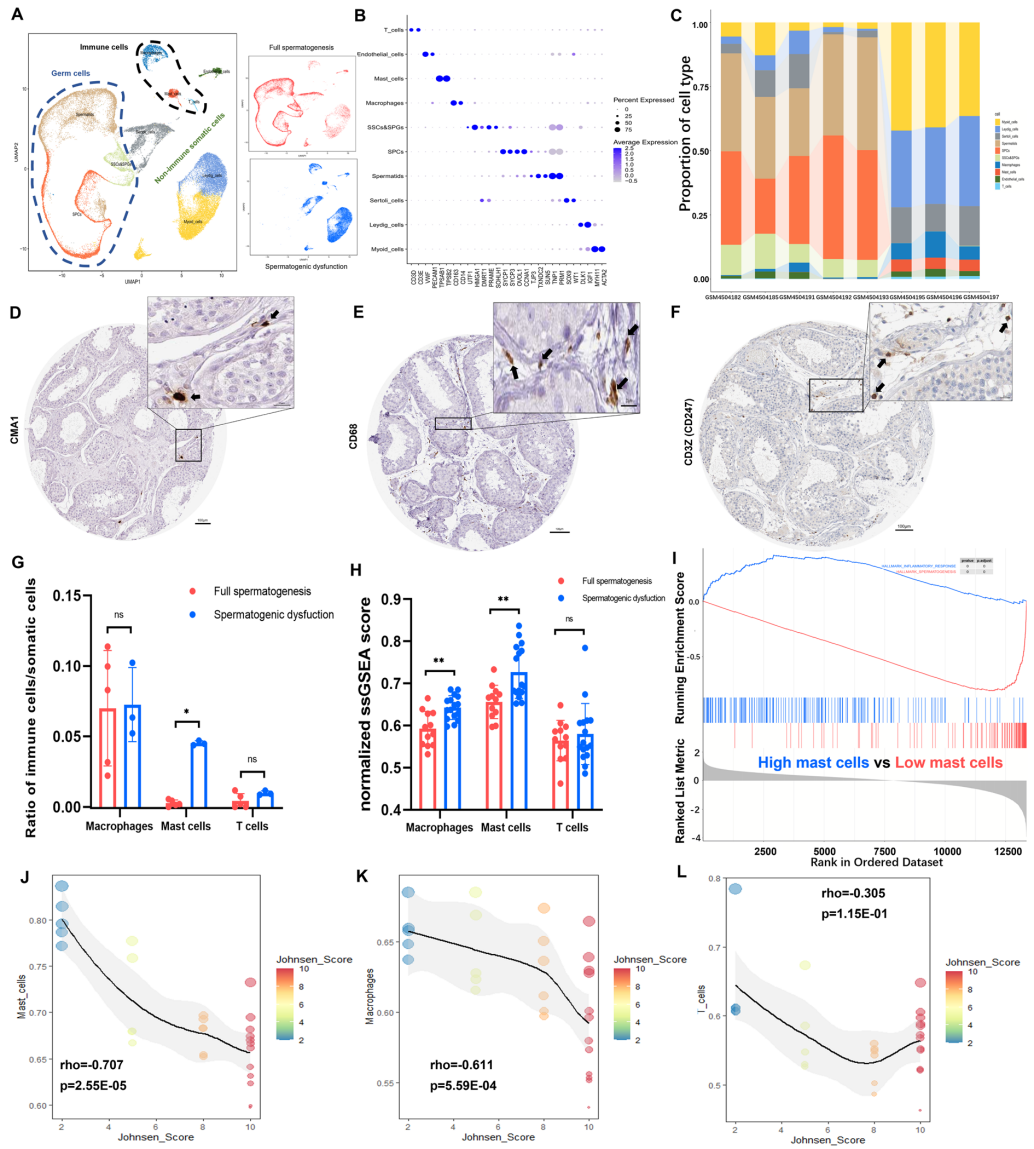
ScRNA-seq and microarray analysis (discovery set) showing main immune cells in the testicular microenvironment of full or dysfunctional spermatogenesis. **A** UMAP plots of cell clusters in all testicular samples as well as in full spermatogenesis or spermatogenic dysfunction group. Cells were colored by different cell types (left) or by different sample groups (right). **B** Dot plot showing expression patterns of canonical marker genes across different cell types. **C** Proportions of all cell types in eight testicular samples. **D**, **E** and **F** Immunohistochemical staining of additional markers of Mast cells (CMA1), Macrophages (CD68) and T cells (CD3Z), respectively, in the testis. Arrows represented positive cells. The original images of IHC stained sections were obtained from Human Protein Atlas database (https://www.proteinatlas.org). **G** Bar plot showing comparison between normal (full spermatogenesis) and disease (spermatogenic dysfunction) samples of the radio of three immune cells to somatic cells. **H** Bar plot showing normalized ssGSEA scores of three immune cells in different groups. Data shown as mean ± SD. *p < 0.05, **p < 0.01, ns: not significant (compared with full spermatogenesis group). **I** The top and bottom enriched pathway (according to NES) in GSEA of differently expressed genes compared between high versus low mast cell infiltration groups. GSEA was based on h.all.v7.4.entrez.gmt. **J**, **K** and **L** Scatter plots showing spearman correlations between JS and mast cells, macrophages and T cells infiltration level, respectively (reflected by ssGSEA scores, similarly hereinafter). rho, spearman correlation coefficient. Dots are colored by different JS. Dot diameter represented immune cell infiltration level. Curves were fitted with loess method. *UMAP* uniform manifold approximation and projection, *ssGSEA* single sample gene set enrichment analysis, *JS* Johnsen score, *NES* normalized enrichment score, *GSEA* gene set enrichment analysis

The testicular immune cell infiltrating landscape was evaluated using scRNA-seq analysis. After filtration, 45,496 cells with 44,453 features per cell were gained for further work. Quality control metrics are shown in Additional file 4: Figure S2. In all, ten cell clusters were manually identified (Fig. 1A) with the expression pattern of cell-type specific or highly expressed marker genes (Fig. 1B) in the integrated eight samples. Germ cells (marked by DDX4) (Additional file 5: Figure S3A, Additional file 6: Figure S4A) were classified into the following three clusters: spermatogonial stem cells and spermatogonia (SSCs&SPGs), spermatocytes (SPCs) and spermatids. Corresponding germ cell markers were successively expressed in accordance with the development of spermatogenesis (Fig. 1B, Additional file 5: Figure S3D–L, Additional file 6: Figure S4D–I). For seven somatic clusters (marked by VIM) (Additional file 5: Figure S3B, Additional file 6: Figure S4B), two subgroups, namely, testicular infiltrating immune cells and non-immune somatic cells, could be divided according to the expression pattern of immune cell marker PTPRC (Additional file 5: Figure S3C, Additional file 6: Figure S4C). The former subgroup contained macrophages, mast cells and T cells (Fig. 1B), while the latter subgroup contained Sertoli cells, Leydig cells, myoid cells as well as endothelial cells (ECs). Macrophages were the largest population of testicular immune cells (Fig. 1A, C and G), followed by mast cells and T cells. Hence, these three types were considered as the three main immune cell types and were further analyzed. For these three immune cell types, additional markers (CMA1 for mast cells, CD68 for macrophages and CD3Z for T cells) were validated in HPA IHC stained sections (Fig. 1D–F). Mast cells showed an increasing trend in the spermatogenic dysfunction group (disease group) (Fig. 1C, Additional file 7: Figure S5). To confirm this phenomenon, we calculated the ratios of each type of immune cells to somatic cells, which demonstrated that the ratio of mast cells was significantly enhanced in spermatogenic dysfunctional samples (p = 0.0357) (Fig. 1G). Macrophages remained the largest immune population in both groups, while the T cell population was small. Thus, these findings suggested that mast cells may play an important role in the development of spermatogenic dysfunction. To confirm this, the discovery set was employed to perform single sample gene set enrichment analysis (ssGSEA) (an algorithm used to overcome the inability of gene set enrichment analysis to evaluate a single sample [18]), using well-known 24 immune cells signatures [19, 20]. The results of the abovementioned three main immune cell types in each sample were extracted (Additional file 15: Table S1). Similarly, the infiltration level of mast cells (reflected by ssGSEA scores, similarly hereinafter) was significantly increased in spermatogenic dysfunction samples (p = 0.0022) (Fig. 1H). Next, all samples of the discovery set were divided into high or low mast cell infiltration groups according to the median value of ssGSEA mast cell scores. Gene set enrichment analysis (GSEA) (a functional enrichment analysis based on given gene sets) [21] was conducted with genes ranked by log2 fold change (logFC) of differential expression analysis between two groups (high versus low). The “spermatogenesis” pathway was significantly enriched in the group with low mast cell infiltration level (normalized enrichment score (NES) = −3.086, adjust p = 1.67E-09, q = 9.47E-10) while the “inflammatory response” was enriched in the high mast cell group (Fig. 1I), confirming that increased mast cell infiltration in the testis may weaken spermatogenic function and strengthen the inflammatory status. Moreover, the mast cell infiltration level showed a significant negative correlation with JS (rho = -0.707, p = 2.55E-05) (Fig. 1J), which further indicated that mast cell infiltration was negatively correlated with testicular spermatogenic function. Therefore, we decided to focus on testicular mast cells. The correlations between the other two immune cell types and JS are also shown in Fig. 1K–L.

### Identifying hub immune genes related to both spermatogenic dysfunction and testicular mast cell infiltration

**Fig. 2:**
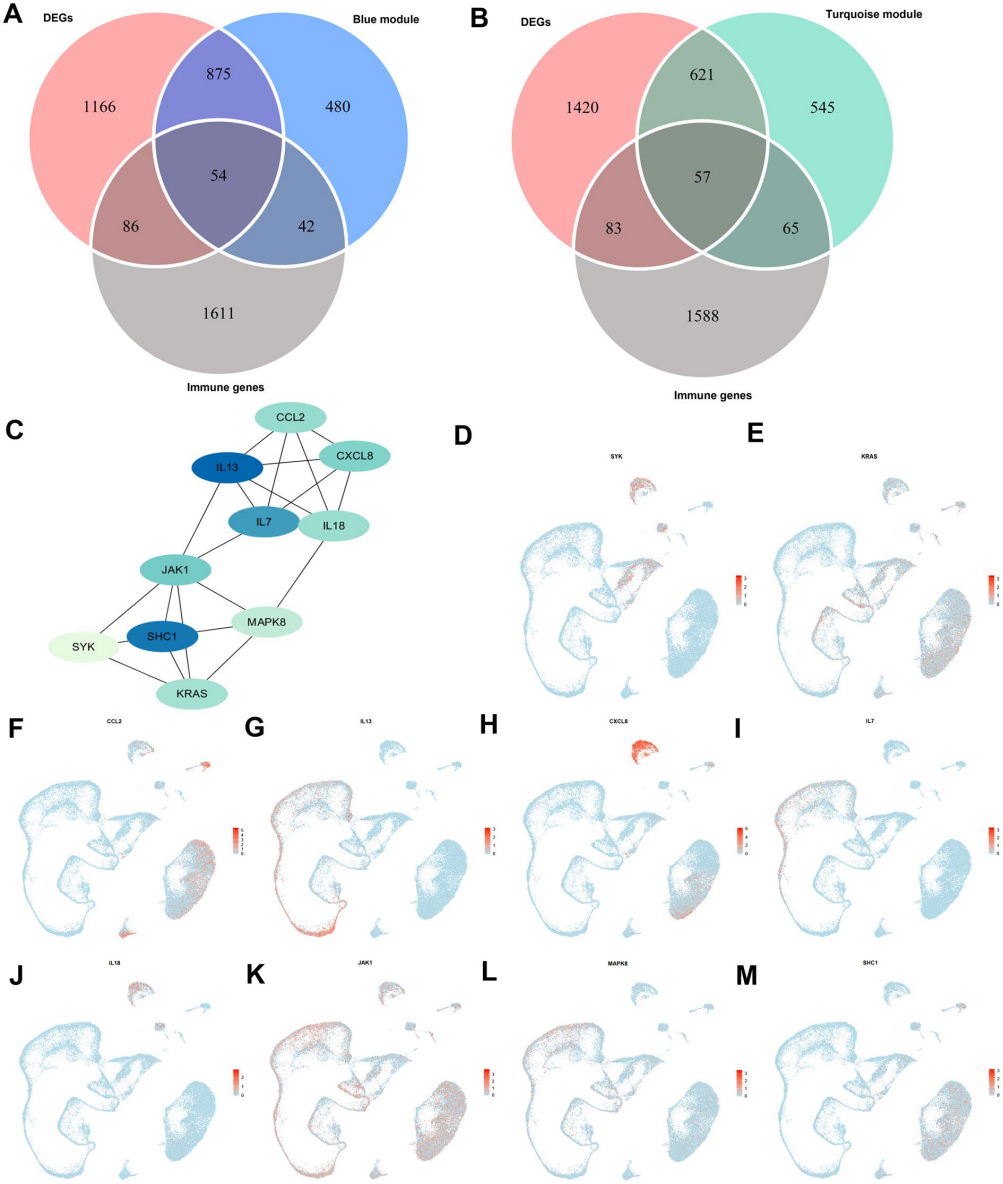
Identification of hub immune genes related to both spermatogenesis and testicular mast cell infiltration (discovery set). **A** and **B** Venn diagrams of DEGs, immune genes intersecting with genes in blue module or turquoise module, respectively. The de-duplicated immune genes list was from ImmPort dataset. **C** PPI network of potential hub immune genes selected by top 10 MCC values. Color intensity of boxes changed positively with the increasing of MCC value. **D**–**M** Expression patterns of 10 potential hub genes in testicular microenvironment. Red color deepened with the expression value of a certain gene. PPI, protein–protein interaction. MCC, maximum clique centrality

We next identified hub testicular genes that had all of the following three characteristics: (1) immune-related genes, (2) genes significantly correlated with testicular mast cell infiltration level and (3) genes significantly correlated with spermatogenic function. A three-step method for identifying such genes was used. First, weighted gene co-expression network analysis (WGCNA) (a method to find synergistic expressed modules of genes) [22] was constructed using the top 5000 genes of the discovery set selected by median absolute deviation (MAD), with soft-threshold power set as 7 and MEDissThres as 0.25 (Additional file 8: Figure S6). Twelve merged modules were created (Additional file 8: Figure S6C) and genes in each module are shown in Additional file 16: Table S2. In brief, the blue and turquoise modules were considered as two key modules because they were strongly related to clinical traits of both mast cell infiltration and spermatogenic function (Additional file 8: Figure S6D), and they showed similar strength of correlations but completely opposite trends. All genes in the two key modules (Additional file 8: Figure S6F and H) were used for Gene Ontology (GO) enrichment analysis and the results are shown in Additional file 8: Figure S6G and I. Second, differentially expressed genes (DEGs) between full spermatogenesis samples (JS = 10) and samples with spermatogenic dysfunction (JS < 10) were identified in the discovery set, and 2181 DEGs were identified (Additional file 9: Figure S7, Additional file 17: Table S3). A Circos plot of the top 100 DEGs and the protein expression pattern of the top five up/down-regulated genes (from HPA) was visualized (Additional file 9: Figure S7B). Down-regulated DEGs were largely related to pathways involved in sperm formation (Additional file 9: Figure S7C–F). While up-regulated DEGs were mainly enriched in pathways like (GO Biological Process, GO-BP) positive regulation of defense/innate immune response (Additional file 9: Figure S7G), (GO Cellular Component, GO-CC) collagen-containing extracellular matrix (Additional file 9: Figure S7H) as well as (Kyoto Encyclopedia of Genes and Genomes, KEGG) Ras signaling pathway (Additional file 9: Figure S7I), which were largely related to immune response. Third, the 1793 deduplicated immune-related genes from the immunology database and analysis portal (ImmPort) were intersected with the DEGs and the genes of two key modules. Altogether, 111 intersected genes were identified (Additional file 18: Table S4), including 54 from immune genes ∩ DEGs ∩ blue module genes (Fig. 2A) and 57 from immune genes ∩ DEGs ∩ turquoise module genes (Fig. 2B). The 111 genes were then used to construct a PPI network and the top ten genes (Fig. 2C) with the highest MCC values were identified as hub immune genes that were related to both mast cell infiltration and spermatogenic function, including CCL2, IL13, CXCL8, IL7, IL18, JAK1, MAPK8, SHC1, SYK and KRAS. The expression pattern of these 10 hub immune genes in testicular cells was visualized using scRNA-seq data (Fig. 2D–M).

### Internal and external validation of hub immune genes

**Fig. 3:**
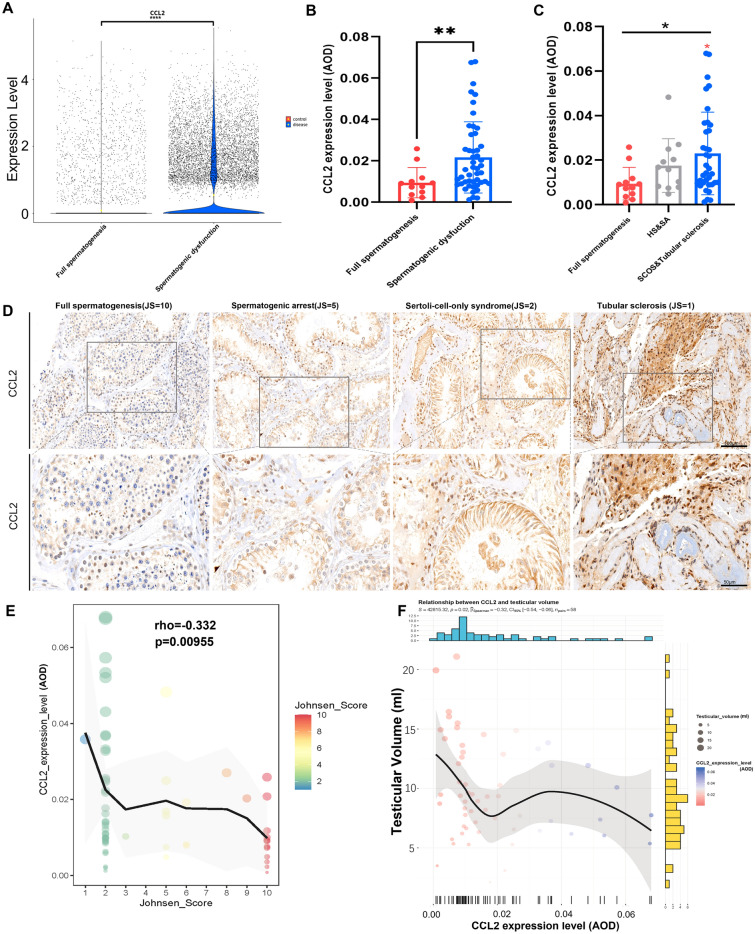
External validation of CCL2 in the testing set. **A** Violin plot of expression level of CCL2 in control (normal spermatogenesis) and disease (spermatogenic dysfunction) groups in scRNA-seq set. Large yellow dots indicated mean expression value, **** adjust p < 0.0001 wilcoxon rank sum test with bonferroni correction using Findmarkers function. **B** Bar plot showing AOD of CCL2 IHC staining in control and disease groups of the testing set. **p < 0.01. **C** Bar plot showing CCL2 level in different testicular pathologic status. Red asterisk represents significant versus full spermatogenesis group. *p < 0.05. **D** Representative CCL2 IHC staining of testes with different spermatogenic status in the testing set. **E** Scatter plots showing spearman correlations between CCL2 expression and JS in the testing set. **F** Scatter plots showing spearman correlations between CCL2 expression and testicular volumes (ml) in the testing set. Note: Only 58 patients were with known testicular volumes from the ultrasound. *AOD* average optical density, *HS* hypospermatogenesis, *SA* spermatogenic arrest, *SCOS* Sertoli-cell-only syndrome

The abovementioned ten genes were tested in both the discovery set (internal validation) and two validation sets (external validations). The expression levels of CCL2, IL13, IL18 and SHC1 showed significant correlations with mast cell infiltration levels in all three sets (all p < 0.05) (Additional file 10: Figure S8B, E and H). In terms of spermatogenic function, the expressions of CCL2, IL13, IL18, MAPK8, SHC1 and KRAS were significantly correlated with JS in the discovery set and both validation sets (all p < 0.05) (Additional file 10: Figure S8C, F and I) (for GSE45885, only the 27 samples in the disease group with clearly marked JS were used here). The relationships among these ten genes are shown in Additional file 10: Figure S8A, D and G. Hence, CCL2, IL13, IL18 and SHC1 passed both internal and external validation and could be considered as hub testicular immune genes that correlated with both mast cell infiltration and spermatogenic function. Afterwards, the Findmarker function (parameter set: logfc.threshold = 0, min.pct = 0.1) was used to examine the expression difference of these four genes in the control and disease groups (scRNA-seq set). IL13 was significantly downregulated in the disease group compared to the control group (Additional file 11: Figure S9A), while CCL2 and SHC1 were significantly upregulated in the disease group (Fig. 3A, Additional file 11: Figure S9B) (all adjust p < 0.0001). IL18 was very infrequently expressed in either group and did not obtain a test result (Additional file 11: Figure S9C), which indicated that IL18 had a less important function in the testis. Because the focus of the present study was the testicular immune microenvironment, key cytokines that could be secreted out to the entire testicular immune microenvironment should be first studied. Therefore, CCL2 and IL13 meet our request. Notably, IL13 was mainly expressed in SPCs and spermatids (Additional file 11: Figure S9D). So, when the spermatogenic dysfunction happens (especially in Sertoli-cell-only syndrome and early spermatogenic arrest), the decrease of IL13 in the microenvironment is most likely due to the decrease of germ cell numbers, rather than the decrease of secretion amount per cell. CCL2, on the other hand, was an important pro-inflammatory chemokine and was upregulated when spermatogenic dysfunction occurred, indicating its more important role in both the testicular immune microenvironment and spermatogenic dysfunction. Consequently, we chose CCL2 as our interested immune hub gene for further verification and study.

### External validation of CCL2 and its relationship with mast cell infiltration in the testing set

**Fig. 4:**
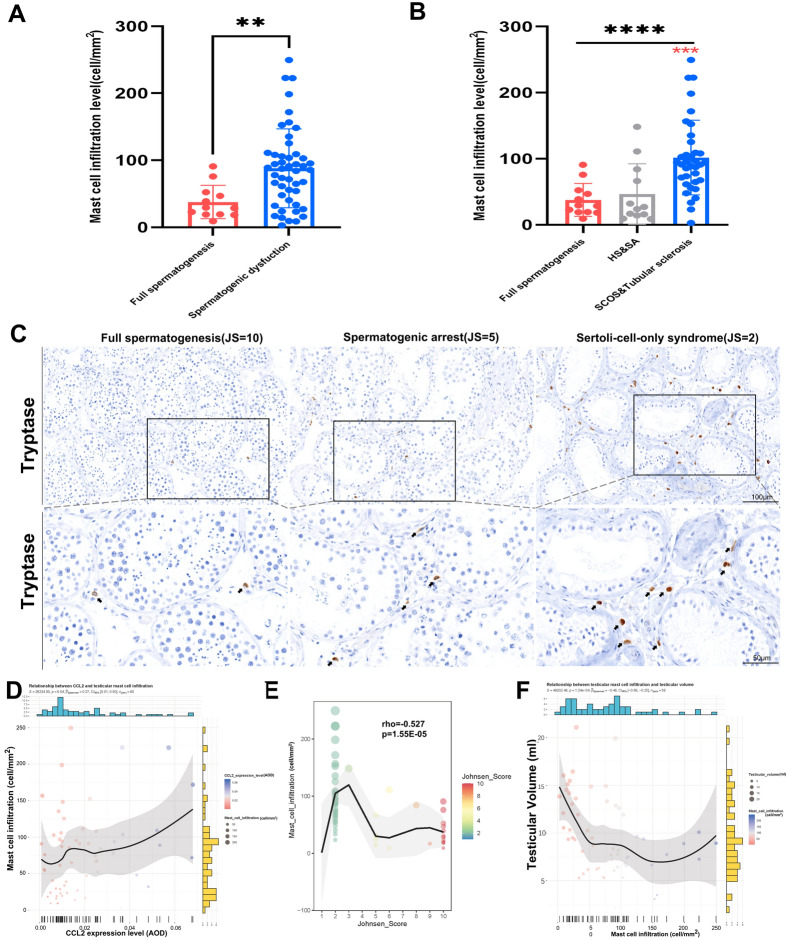
External validation of mast cell infiltration in the testing set. **A** Bar plot showing IHC staining of mast cell (Tryptase) infiltration level (cell/mm^2^) in control and disease groups of the testing set. **p < 0.01. **B** Bar plot showing IHC staining of mast cell (Tryptase) infiltration level (cell/mm^2^) in different testicular pathologic status. Red asterisk represents significant versus full spermatogenesis group. ****p < 0.0001; ***p < 0.001. **C** Representative mast cell (Tryptase) IHC staining of testis with different spermatogenic status in the testing set. **D** Scatter plots showing spearman correlations between CCL2 expression and mast cell infiltration level in the testing set. **E** Scatter plots showing spearman correlations between mast cell infiltration level and JS in the testing set. **F** Scatter plots showing spearman correlations between mast cell infiltration level and testicular volumes (ml) in the testing set. Note: Only 58 patients were with known testicular volumes from the ultrasound. *AOD* average optical density, *HS* hypospermatogenesis, *SA* spermatogenic arrest, *SCOS* Sertoli-cell-only syndrome

In both the discovery set and validation sets, CCL2 was negatively correlated to spermatogenic function (Additional file 12: Figure S10B, D and F). To confirm this pattern, we used the testing set (Additional file 3: Figure S1E) for further external validation. The testing set contained 60 testicular samples donated by azoospermia patients at our institute, including 48 from NOA patients (as the spermatogenic dysfunction group) and 12 from obstructive azoospermia (OA) patients with pathological confirmation of full spermatogenesis (as the full spermatogenesis group). The clinicopathological features of the testing set are shown in Additional file 3: Figure S1A–D. IHC staining of CCL2 in the testing set (Fig. 3D) confirmed that CCL2 expression was significantly upregulated in spermatogenic dysfunctional samples (p = 0.0072) (Fig. 3B), especially in severe pathological status (Fig. 3C), which was in line with results of the scRNA-seq set. Moreover, the CCL2 expression level was confirmed to be significantly negatively correlated with JS (p = 0.0096) (Fig. 3E) and testicular volumes (p = 0.015) (Fig. 3F). Regarding mast cells, CCL2 was found to be significantly positively correlated to testicular mast cell infiltration (Additional file 12: Figure S10A, C and E). We also validated these findings in the testing set (Fig. 4C) and found that mast cell infiltration level was significantly upregulated in spermatogenic dysfunctional testes(p < 0.01) (Fig. 4A), especially in severe pathological conditions (Fig. 4B). The mast cell level was also confirmed to be significantly positively correlated with CCL2 expression (p = 0.036) (Fig. 4D) or negatively correlated to JS (p < 0.0001) & testicular volumes (p = 0.00012) (Fig. 4E–F), which was all identical to the results in the discovery and validation sets.

### Function of CCL2 in spermatogenic dysfunction, mast cell infiltration and the testicular immune microenvironment

**Fig. 5:**
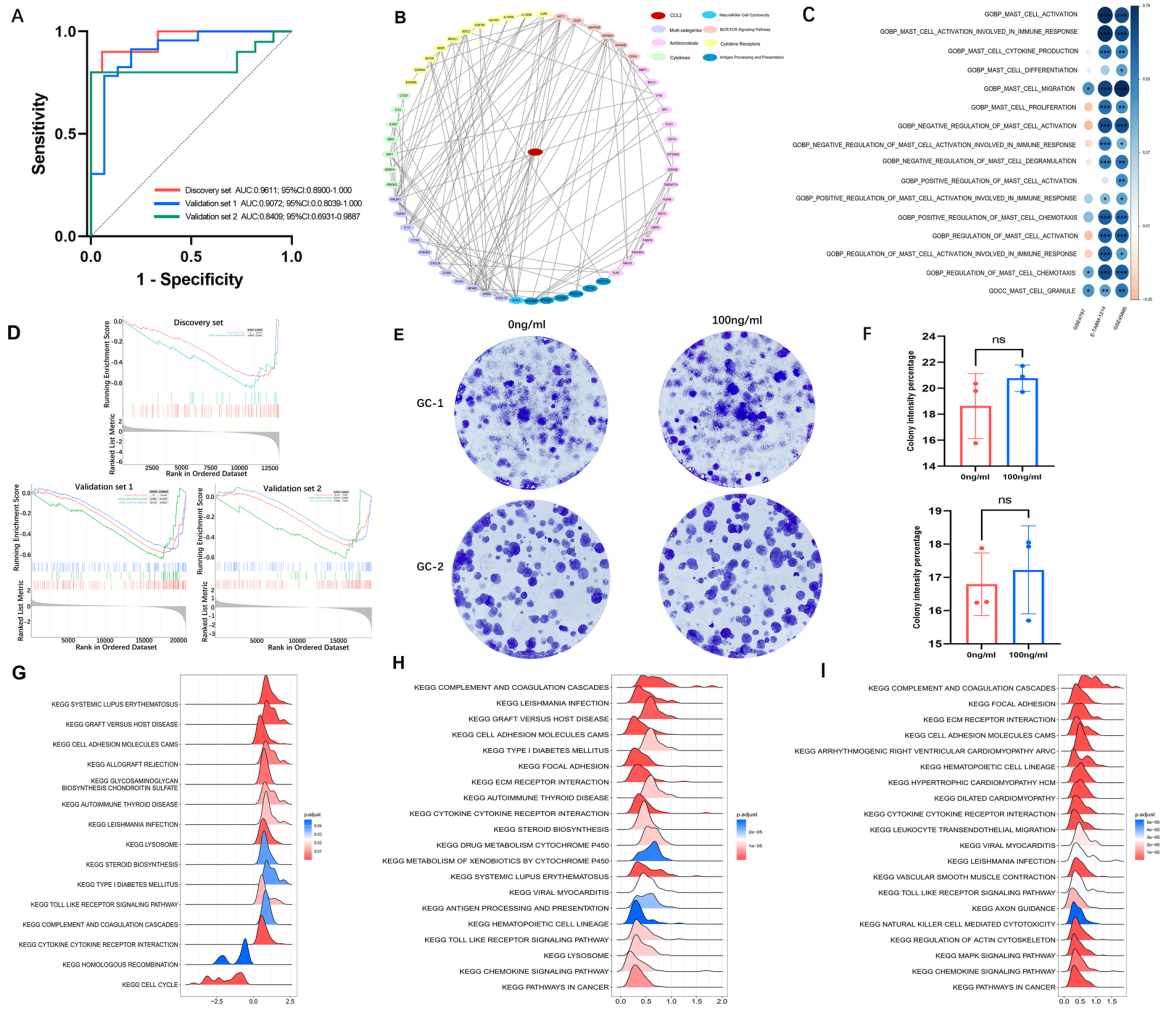
The roles of interested hub immune gene CCL2 in clinical predicting, testicular immune microenvironment, testicular mast cell regulation and spermatogenic dysfunction. **A** ROC curves of CCL2 for predicting the success of TESE surgery (samples with JS/mJS ≥ 8 versus JS/mJS < 8). **B** CCL2-related immune PPI network in the testis. Different colors represented the gene categories summarized from the original immune gene list. **C** Heatmaps of spearman correlations between GSVA scores of mast cell-related signatures and CCL2 expression level in the discovery and both validation sets. Blue and red represented positive and negative correlations, respectively. The color intensity and spot diameter indicate |correlation coefficient|. *p < 0.05, **p < 0.01, ***p < 0.001. **D** Negatively enriched pathway (NES < 0) in GSEA of differently expressed genes compared between high versus low CCL2 groups in spermatogenic dysfunctional samples of the discovery set, validation set1 and validation set2. GSEA was based on c2.cp.kegg.v7.4.entrez.gmt. **E** In vitro colony formation experiments showing colony formation ability of GC-1 (up) and GC-2 cells (down) with (100 ng/ml) or without (0 ng/ml) Ccl2 treatment. **F** Bar plots showing CIP of the colony formation experiments for GC-1 (up) and GC-2 (down) cell lines. ns, not significant. **G**, **H** and **I** Ridge plots of the top 20 KEGG pathways (according to p values) enriched in GSEA analyses comparing high versus low CCL2 expression levels in samples with spermatogenic dysfunction (discovery set, validation set 1 and validation set 2, respectively). Note: there were only in total 15 pathways enriched in the discovery set. *ROC* receiver operating characteristic, *TESE* testicular sperm extraction, *GSVA* gene set variation analysis, *CIP* colony intensity percentage

Based on the fact that JS/mJS ≥ 8 indicated the existence of testicular spermatozoa[3, 4] (also called mature spermatids) [4], we used receiver operating characteristic (ROC) curves for differentiating testicular samples that could attain successful sperm retrieval from samples without spermatozoa. The area under the curves (AUCs) were all  > 0.8 (Fig. 5A), indicating CCL2 might be used as an effective biomarker to predict sperm retrieval in testicular sperm extraction (TESE). Moreover, the CCL2-related testicular immune PPI network showed a tight relation of CCL2 with various testicular expressed immune genes (Fig. 5B). Gene set variation analysis (GSVA) (an algorithm for calculating the enrichment statistic of each gene set in each sample) [23] of both the discovery and validation sets were conducted with 16 mast cell-related signatures (Additional file 13: Figure S11). In addition, spearman correlation analyses between CCL2 expression and calculated GSVA scores of each gene signature were performed. The CCL2 expression level was significantly positively related to mast cell migration, regulation of mast cell chemotaxis and mast cell granule (p < 0.05 among three datasets) (Fig. 5C), which indicated that the increase of testicular mast cells related to CCL2 may be caused by the recruitment of mast cells from the circulatory system. To detect the mechanisms of CCL2 involved in spermatogenic impairment and mast cell enrichment, spermatogenic dysfunction samples of the discovery set and validation sets were used for GSEA based on the KEGG set according to the abovementioned GSEA methods. The diminishing pathways in the high CCL2 group (NES < 0) were mainly related to cell cycle and meiosis (Fig. 5D, Additional file 19: Table S5), which suggested that CCL2 in the testicular microenvironment may correlate with the disorder of the cell cycle of spermatogenic cells. Nevertheless, the addition of Ccl2 protein to spermatogenic cell lines (GC-1 or GC-2 cells) did not significantly change the colony formation ability of either cell line (Fig. 5E–F), which suggested that CCL2 might lead to spermatogenic dysfunction in an indirect manner. Importantly, most of the pathways were enriched in the high CCL2 group (NES > 0). Ridge plots showed the top 20 enriched pathways (according to p values) in disease samples of the discovery set and both validation sets (Fig. 5G–I). Five pathways, including “cytokine-cytokine receptor interaction”, “complement and coagulation cascades”, “cell adhesion molecules cams”, “toll like receptor signaling pathway” and “leishmania infection”, were the common pathways. These results agreed with the well-known function of CCL2 as a crucial cytokine that interacts with its receptors to play roles [24].

### CCL2-mediated cytokine-cytokine receptor interaction and cell–cell communications in the testicular microenvironment

**Fig. 6:**
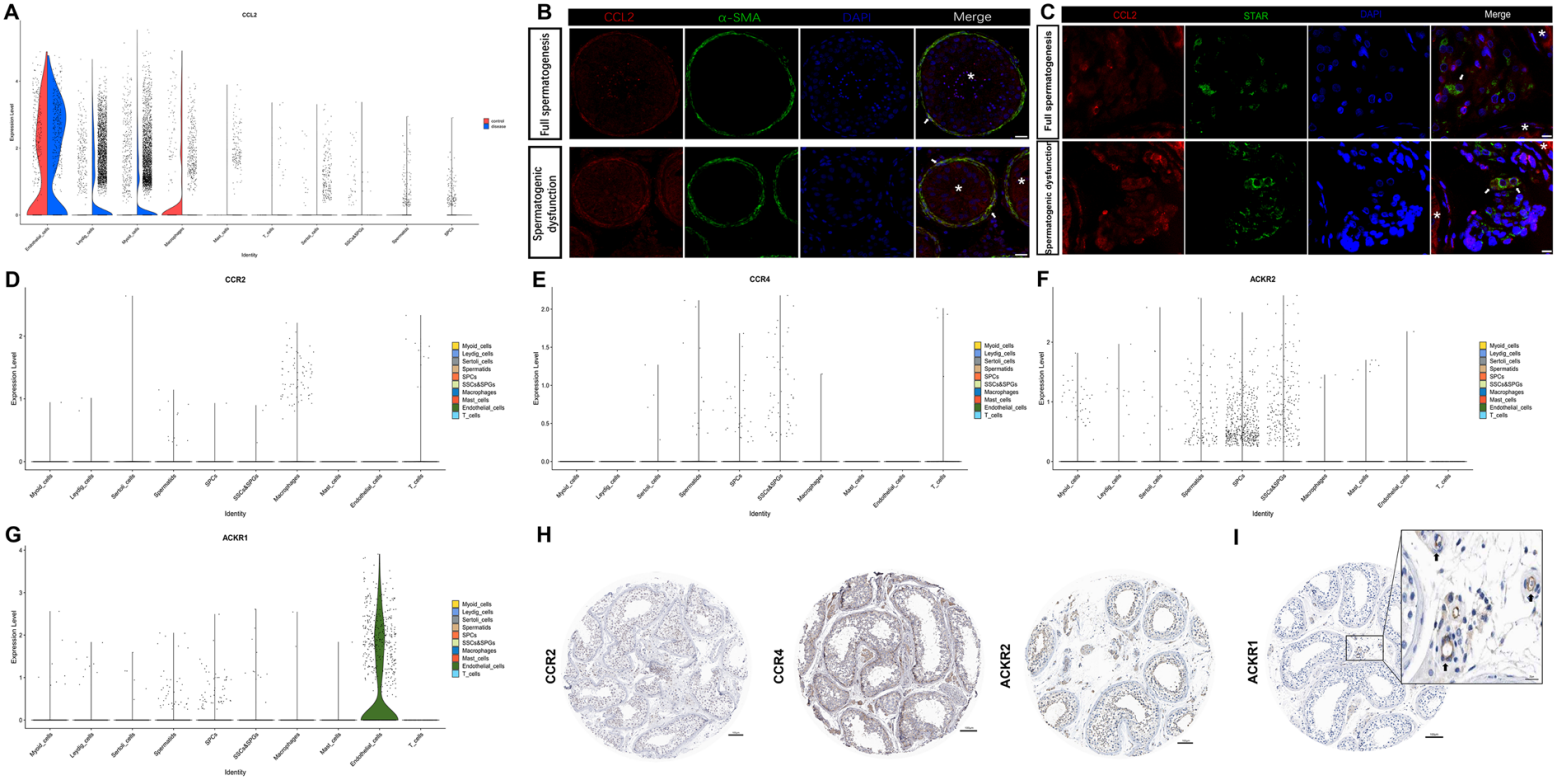
Expression pattern of CCL2 and its receptors in the testis **A**Violin plots showing expression level of CCL2 among different cell types in the testis. The violin plots were split by groups. **B**, **C** Immunofluorescence staining of CCL2 (red) and α-SMA (green, 6B) or STAR (green, 6C). Nuclei were stained with DAPI dye (blue). * represent seminiferous tubule. Arrows represent typical myoid cells (6B)/Leydig cells (6C) of the fields. The scale bars represent 20 μm (in 6B) and 10 μm (in 6C). **D**, **E**, **F** and **G** Violin plots showing the expression pattern of CCL2 receptors CCR2, CCR4, ACKR2 and ACKR1, respectively, among different cell types in the testis. **H** and **I** Immunohistochemical staining of CCR2/CCR4/ACKR2 and ACKR1, respectively, in the testis. Arrows represented obvious positive cells. The original images of IHC stained sections were obtained from Human Protein Atlas database (https://www.proteinatlas.org)

**Fig. 7:**
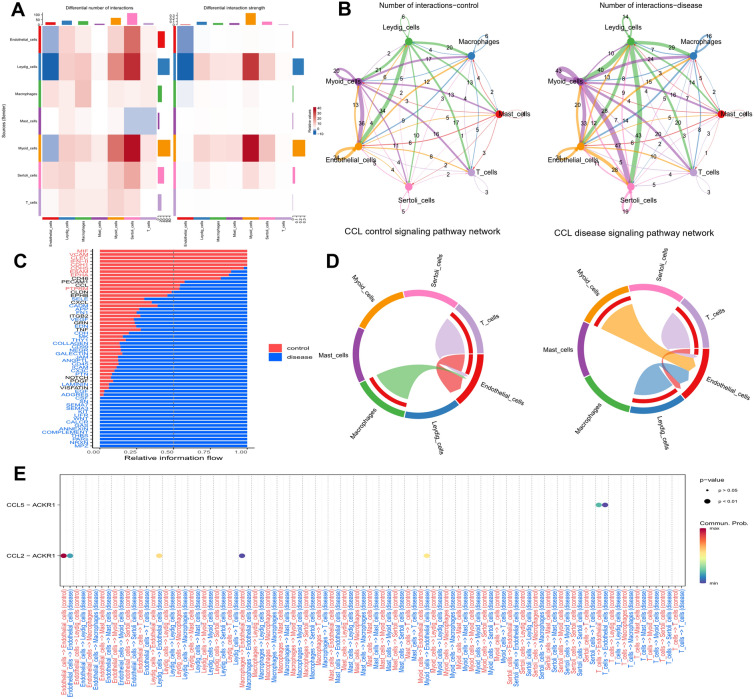
Cell–cell communications among somatic cells in the testicular microenvironment of control and disease groups. **A** Heatmaps of different interaction numbers (left) and strength (right) showing the comparison between disease and control groups. Red color or blue color represented upregulation or downregulation (respectively) in the disease group compared to the control group. **B** Network diagrams of counts of somatic cell–cell interactions in control (left) and disease (right) groups. The size of lines and numbers on lines indicated counts of interactions. Arrows reflected the direction of signals from one cell type to another. **C** Comparison of strength of different pathways between control and disease group. Names in red or blue indicated pathway was significant strengthened in control or disease groups, respectively (paired Wilcoxon test, p < 0.05 as significant). Names in black meant no significant difference between groups. **D** Chord plots reflecting CCL signal pathway networks in control (left) and disease (right) groups. **E** Bubble plot of detailed CCL signal pathways in cell–cell chats

Since CCL2 was expressed in the testis, we then explored which type of testicular cells might be the sources of testicular CCL2. As shown in Fig. 6A, CCL2 maintained a high average expression level in ECs in both the control and disease groups (adjust p > 0.05 between groups) but sharply shrank in macrophages of the disease group. Unfortunately, the proportions of both EC (average 1.7%) and macrophages (average 3.5%) in testicular cells are rather low, which indicated that they may not be responsible for the main sources of testicular CCL2. On the other hand, myoid cells and Leydig cells showed an increasing trend of CCL2 in the disease group (both adjust p < 0.0001) and the numbers of these two cells were quite large (average 18.2% and 14.4%, respectively). The expression of CCL2 in myoid and Leydig cells were also shown by IF staining (Fig. 6B–C). As for germ cells, although some of the SPCs and spermatids showed CCL2 expression, they did not have that high expression level. Interestingly, Sertoli cells were found to have a rather strong expression of CCL2 according to IF staining (data not shown) but did not show a high level of expression in the scRNA-seq set. Such contradictions need to be investigated further. Therefore, in both the scRNA-seq data and IF data, we confirmed the sources of testicular CCL2 could be at least partially attributed to myoid cells as well as Leydig cells, especially in spermatogenic dysfunction. So, we chose these two CCL2-expressing cell types to do further analysis. Unexpectedly, the expressions of two conventional CCL2 receptors (CCR2 and CCR4) and one atypical receptor (ACKR2) were not abundant in the testicular microenvironment (Fig. 6D–F). At protein level, both CCR2 and CCR4 were recorded as not detected/low expressed (Fig. 6H, protein expression information from HPA database), and ACKR2, although showed medium protein level in some of the germ cells (Fig. 6H, protein expression information from HPA database), might suffer from reductions due to the loss of germ cells when spermatogenic dysfunction happened. Therefore, we deduced that these three receptors (CCR2/CCR4/ACKR2) might be less important during cell-cell communications in spermatogenic dysfunction of human testes. Another atypical CCL2 receptor ACKR1 had an abundant expression level among testicular ECs (Fig. 6G, I). Therefore, these findings suggested that “myoid/Leydig cells-CCL2-ACKR1-ECs” axis might be one of the CCL2-mediated cytokine-cytokine receptor interaction pathways in the testicular microenvironment, especially in spermatogenic dysfunctional testes. To further confirm this hypothesis and to explore cell–cell communication network in the testicular microenvironment, the CellChat package was employed. Due to the lack of spermatogenic cells in the disease group, the CellChat analysis was based only on testicular somatic cells to compare the two groups. The heatmaps of the differential number of interactions and interaction strength are shown in Fig. 7A. The numbers of interactions between myoid/Leydig cells and ECs were slightly decreased in the disease group (Fig. 7B). Regarding the interaction strength, the CCL signaling pathway was not significantly different between the normal spermatogenic group and the spermatogenic dysfunction group (Fig. 7C). These results may be due to the following two explanations: (1) the CCL signal did not change; and (2) the CCL signal was enhanced in the communication between some types of cells but decreased in others, which did not change the total information flow. In particular, we found the CCL signal information flow between myoid/Leydig cells and ECs was significantly enhanced in the disease group (Fig. 7D), while the CCL signal information flows between macrophages and ECs as well as EC-EC self-communication were significantly downregulated (Fig. 7D). This finding suggested that the second explanation was correct. Specifically, the communication probability of the CCL2-ACKR1 signal from myoid/Leydig cells to ECs was enhanced in the spermatogenic dysfunction group, while the remaining CCL signals were all downregulated (Fig. 7E). Overall, these findings revealed that the “myoid/Leydig cells-CCL2-ACKR1-ECs” axis was an important CCL2-mediated cytokine-cytokine receptor interaction pathways in spermatogenic dysfunction.

### Testicular mast cell subtypes and its relationship with endothelial cells

**Fig. 8:**
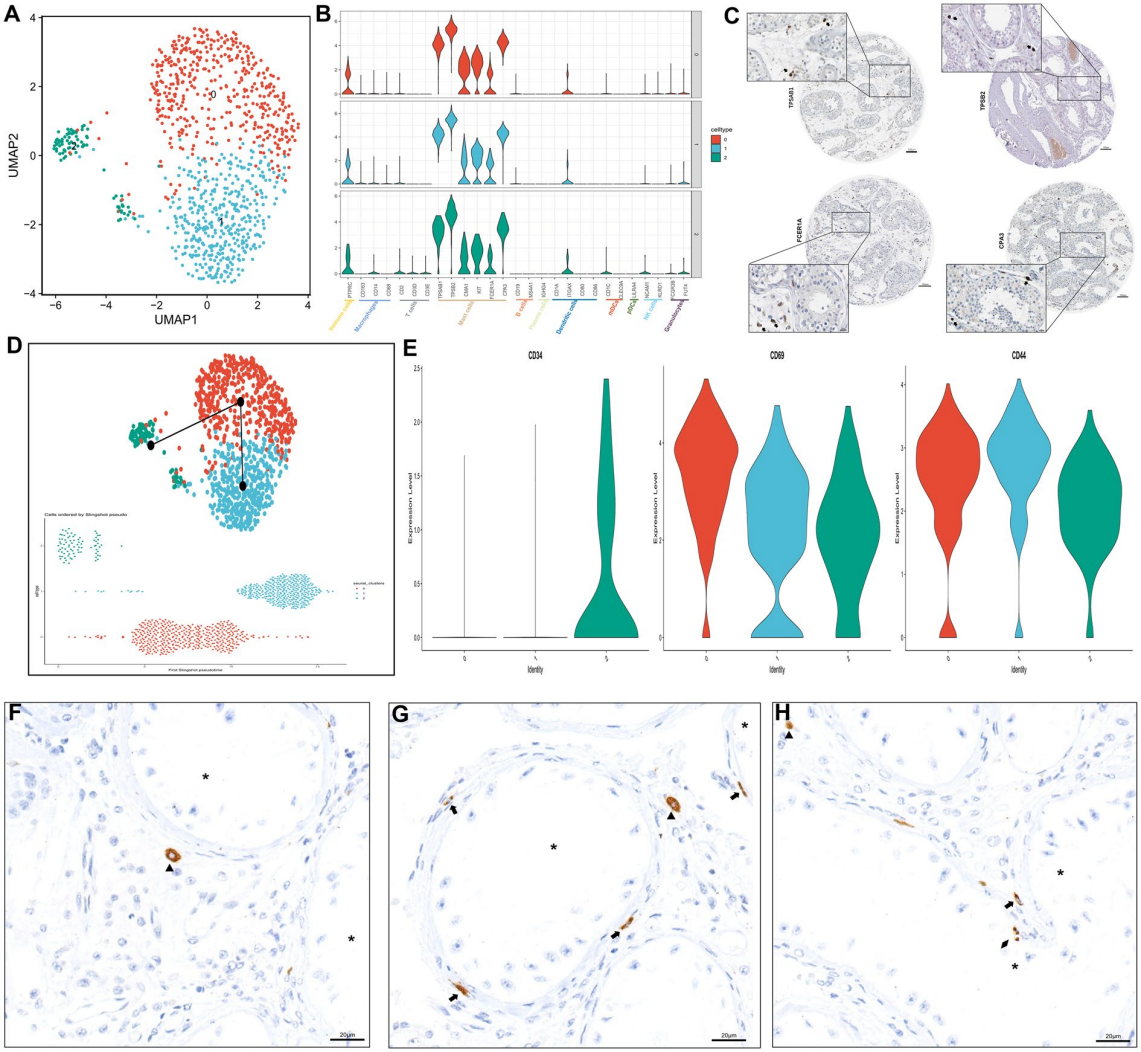
Subtypes of testicular mast cells in the spermatogenic dysfunctional testis. **A** UMAP plots of mast cells in the disease group. Cells were colored by subclusters. **B** Validation of mast cell markers and markers of other types of immune cells among subcluster 0–2. **C** IHC staining of mast cell markers including TPSAB1, TPSB2, FCER1A and CPA3 in the testis. Arrows represented positive cells. The original images of IHC stained sections were obtained from Human Protein Atlas database (https://www.proteinatlas.org). **D** UMAP plots with trajectory lineage of testicular mast cells (up) and pseudotime analysis of mast cell cluster 0–2 using slingshot (down). **E** Violin plots showing the expression pattern of CD34, CD69 and CD44 in three mast cell subclusters. **F**, **G** and **H** IHC staining showing different distribution of mast cells. Triangles represented interstitial mast cells (located in testicular interstitium). Arrows represented peritubular mast cells (surrounding or presented in tubular walls). Diamond represented (extremely rare) mast cells breaking through basal lamina. * represented lumens of seminiferous tubules. Note that due to the existence of few mast cells in normal samples, we only do re-cluster of mast cells in disease samples of scRNA-seq set

Next, we re-clustered mast cells from disease samples and identified three subclusters based on a resolution of 0.2 (Fig. 8A). Mast cell markers, including TPSAB1, TPSB2, CMA1, KIT, FCER1A and CPA3, as well as the immune marker PTPRC, were highly expressed among the three clusters, while markers of macrophages, T cells, B cells, plasma cells, dendritic cells (DCs), mDCs, pDCs, NK cells or granulocytes were all expressed at low levels (Fig. 8B), reconfirming that the three subclusters were all mast cells. The expression of the mast cell markers was also shown by HPA IHC staining (Figs. 8C, 1D and Additional file 5: Figure S3E). Only one lineage was identified in trajectory inference (Fig. 8D), and pseudotime analysis found that testicular mast cells started from cluster 2, then moved to cluster 0, and finally reached cluster 1 (Fig. 8D). We found that CD34, a typical marker of hematopoietic progenitor cells [25], was basically expressed in subcluster 2 (Fig. 8E). Combined with the results in Fig. 8B, cluster 2 was comprised of Lin(-)/CD34( +)/CD117 ^int/hi^/FcεRI( +) cells, which indicated that this cluster might be mast cell progenitors [26–28]. CD69, a leukocyte early-activation molecule [29, 30], was significantly upregulated in cluster 0 (Fig. 8E). And CD44, which was upregulated in differentiated and mature mast cells [31], was higher in cluster 1 (Fig. 8E). These results were consistent with the pseudotime analysis, which indicated that cluster 2 might be mast cell progenitors, and that cluster 1 might be finally maturated mast cells. In terms of the distribution of testicular mast cells, all mast cells could be basically divided into three types, including interstitial mast cells (located in the testicular interstitium and had a rounder shape) (Fig. 8F), tubular wall mast cells (surrounding or in the tubular wall and had a slender shape) (Fig. 8G) and invasive mast cells (appearing at the inner surface of basal lamina, this type was rare and its existence need to be further confirmed) (Fig. 8H). Subsequently, the relationship between ECs and mast cells was further studied. Briefly, the average gene expression of ECs in scRNA-seq samples was calculated by AverageExpression with zero-expressed genes removed. The GSVA package was then employed to calculate the scores of signatures in the MSigDB collection category “C5” and results between disease and control samples were compared by the limma package. Gene signatures containing the keyword “MAST_CELL” were selected for analysis (Additional file 14: Figure S12A). The “positive regulation of mast cell chemotaxis” and “regulation of mast cell chemotaxis” pathways were significantly activated among ECs in spermatogenic dysfunction samples (adjust p < 0.05). It demonstrated that ECs might be responsible for recruiting mast cells into the testicular microenvironment when spermatogenic impairment occurred. Significantly, ECs might simultaneously express SELE and ACKR1, which were also known as two venous EC makers [32] (Fig. 6G and Additional file 14: Figure S12C). Evidence of SELE protein existing in testis was also shown in Additional file 14: Figure S12B using the HPA IHC section (although not strong, which might need further confirmation). SELE could mediate leukocyte tethering and rolling interactions on ECs, thus enabling leukocytes to bind to and seep into tissue [33]. Previous studies have shown that SELE could trigger mast cells adhering to ECs and diapedesis [34, 35]. Therefore, we inferred testicular ECs might be a bridge, of which one end is attached to CCL2 using ACKR1, and another end is fixed to mast cells using SELE, especially in spermatogenic dysfunctional testes. In addition, the SELE signal information flow between mast cells and ECs was upregulated in the disease group (Additional file 14: Figure S12E). In particular, the testicular EC SELE contact with mast cell CD44 and the communication probability of the “ECs-SELE-CD44-mast cells” axis was strengthened when spermatogenic dysfunction occurred (Additional file 14: Figure S12F). Altogether, these findings suggested that testicular ECs might play a connecting role by linking CCL2 to mast cell infiltration in spermatogenic dysfunctional testes. And the results preliminarily showed a potential “Myoid/Leydig cells-CCL2-ACKR1-ECs-SELE-CD44-Mast cells” communication network in the microenvironment of testes with spermatogenic dysfunction.

## Discussion

Immune cells are considered a two-edged sword in the testicular microenvironment [8, 36, 37]. Although many studies have revealed different potential biomarkers of spermatogenic dysfunction [38–41], few of these biomarkers are immune genes, and their relationship with testicular immune cells remains unclear. In the present study, we focused on testicular mast cells, and for the first time identified and validated the immune biomarker CCL2, which was correlated with both the mast cell infiltration level and the spermatogenetic function. Moreover, we preliminarily drew the potential CCL2-related networks of cell–cell communications in the testicular microenvironment.

Mast cells exist in the microenvironment of both normal testes and spermatogenic dysfunctional testes [10]. In our study, we found that the mast cell infiltration level was significantly negatively correlated with JS, which further suggested the important role of mast cells in spermatogenic dysfunction. Moreover, the testicular mast cells in spermatogenic impaired samples expressed both tryptase (coded by TPSAB1 and TPSB2) and chymase (coded by CMA1), which was in accordance with a previous study [9]. Chymase could induce remodeling of the extracellular matrix and activation of pro-inflammatory cytokines, such as IL-1β and IL-6 [42], which might damage spermatogenic function, causing a lower JS. Notably, in spermatogenic dysfunctional testes, we identified a small cluster which might be mast cell progenitors, suggesting that the testis could recruit mast cell progenitors to the microenvironment and then complete maturation. A previous study has reported that mast cells are present in the tubular lumen in spermatogenic dysfunctional testes [10]. But in our testing set, we only found mast cells that clung to the inner surface of tubular walls without moving to the inside of lumen. Hence, further studies are required to determine whether mast cells could shift to the inside of the tubular lumen.

Testicular CCL2 has been reported to be correlated to hypogonadism and is upregulated in testes of mice with metabolic syndrome [43]. The present study demonstrated a negative correlation between testicular CCL2 and JS of human testes. Although high level of CCL2 was strongly associated with interruption of the cell cycle of germ cells, this protein itself might not be able to directly kill spermatogonia or spermatocytes, indicating that CCL2 affects spermatogenesis in an indirect manner. Wang et al. revealed that adding Ccl2 could directly damage murine BTB’s integrity [44]. We assume that CCL2 might directly weaken and open the BTB, leading to the entrance of various harmful factors into the tubular lumen, causing the impair of germ cells as well as disorder of the spermatogenic microenvironment. It is worth mentioning that in validation set 2, the four control samples (although called “full spermatogenesis”) came from commercially available total RNA from normal testicular tissue, which indicated that they were not pathologically evaluated with JS by the authors. Therefore, we removed these four samples when performing analyses regarding JS. We found that in somatic cells, CCL2 seemed to be expressed, especially in myoid cells, Leydig cells and ECs. IF staining also demonstrated the potential CCL2 expression in Sertoli cells (data not shown), but this phenomenon was not obviously shown by scRNA-seq data. Such inconsistency might be attributed to the issue of sequencing depth or interindividual variations, thus requiring future studies. In the current study, based on this contradiction, we chose not to analyze Sertoli-cell-expressing CCL2 in depth.

As an immune chemokine, the canonical function of CCL2 is “macrophage chemotaxis”. Besides, CCL2 may have a chemotactic effect on immune cells other than macrophages, including T cells [45] and mast cells [46]. Such chemotactic effects are usually CCR2-dependent. However, we showed that testicular mast cells lacked the expression of conventional CCR2 and CCR4, indicating that testicular CCL2 correlated with mast cell infiltration in an indirect manner. ACKR1, an atypical receptor of CCL2, was highly expressed on testicular ECs, and CCL2-ACKR1 interactions between myoid/Leydig cells and ECs were enhanced in spermatogenic dysfunction testes. Previous studies found that ACKR1 expressed on ECs could cause chemokine internalization and transcytosis, which then triggered the appearance of inflammatory chemokines on ECs [47–49]. Chemokine transcytosis by ACKR1 could lead to apical retention of undamaged chemokines and increase transendothelial migration of leukocytes [49]. Accordingly, we also found that in the spermatogenic dysfunction group, “positive regulation of mast cell chemotaxis” of testicular ECs were significantly upregulated. Therefore, these findings suggested that ECs might act as a bridge, of which one end is linked with the increased testicular CCL2 level and another end is linked with mast cells in blood vessels. To date, there seems to be no research focusing on human testicular ECs, indicating that additional studies are required on testicular ECs and their roles in regulating CCL2 and mast cells. A previous study has revealed that binding of endothelial selectins to mast cell ligands might be responsible for the infiltration of mast cells in inflammatory locations, in which mast cells could complete transendothelial movement [50]. We further found that the interaction between EC SELE and mast cell CD44 (one of the important E-selectin ligands [51]) was enhanced, indicating that CD44 might be responsible for the enhancement of mast cell number. Thus, the present study figured out a potential cell–cell cross-talk network of “Myoid/Leydig cells-CCL2-ACKR1-ECs-SELE-CD44-Mast cells”, which might play a role in the testicular immune microenvironment of spermatogenic dysfunction. More studies with larger cohorts should focus on this network and interpret the function of this network in the changes of spermatogenic function.

Some limitations of this study could not be ignored. First, the patient cohorts in this study were small, indicating that large-scale validations are needed to confirm our results. Second, the limited number of biological replicates used for IF staining made it difficult to conduct statistical analyses of between-group comparisons, and the results may be influenced by individual variations. Hence, additional samples as well as different research methods (such as flow cytometry) should be used in future studies. Third, the testing set only included patients with the normal karyotype and no Y chromosome microdeletion. Thus, it remains unknow whether there is a difference in those azoospermia patients with abnormal chromosomes. More studies are required to focus on such special patients.

## Conclusions

The present study verified CCL2 as an immune biomarker related to both testicular mast cell infiltration and spermatogenic dysfunction. Moreover, a potential “Myoid/Leydig cells-CCL2-ACKR1-ECs-SELE-CD44-Mast cells” network of somatic cell–cell communications in the testicular microenvironment was preliminarily determined. The present findings partially revealed the immunomodulation of testes, providing new diagnostic and therapeutic targets for spermatogenic impairment, especially for NOA.

## Methods

### Single-cell RNA sequencing data collection, processing, integration and clustering analysis

The scRNA-seq data of GSE149512 were employed [15], and only OA patients with normal spermatogenesis (five samples, control/full spermatogenesis group) and idiopathic NOA patients (three samples, disease/spermatogenic dysfunction group) were included. UMI count tables of these eight patients were downloaded from the GEO database and were loaded into the R Studio server (v4.1.1). Seurat objects were created using the Seurat package (v4.1). According to the original article [15], the eight samples belonged to four batches. Hence, samples within one batch were first created as one single Seurat object. Cells were further filtered and data were normalized based on the same methods used in the original article [15]. IntegrateData function was used to do batch correction among four batches and the merged Seurat object was then scaled by the ScaleData function. Principal components (PCs) were obtained using the RunPCA function. Thirty PCs were employed to perform FindNeighbors analysis and uniform manifold approximation and projection (UMAP). FindClusters function with a resolution of 0.2 was used to cluster all cells. The canonical and newly reported marker genes were used to identify the cell types of each cluster [13–17]. The ggalluvial (v0.12.3) package was employed to illustrate the proportion of each cell type in each scRNA-seq sample. The FindMarkers function was used to explore DEGs of scRNA-seq data between clusters or groups. The Cell–cell communication network was analyzed using the CellChat package (v1.1.3). Gene set variation analysis(GSVA) for scRNA-seq data was performed using the GSVA package (v1.40.1) [23] based on MSigDB collection category “C5” retrieved by the msigdbr package (v7.4.1). GSEA [21] for scRNA-seq data was conducted with the clusterProfiler package (v4.0.5) [52, 53]. For re-clustering a certain cell type, UMI raw counts of these cells were extracted and the abovementioned scRNA-seq data process was repeated. Trajectory inference and pseudotime analysis of cells were conducted with the Slingshot package (v2.0.0) [54].

### Patients and tissue samples

Testicular tissue samples used in this study were donated by azoospermia patients who underwent TESE, microdissection TESE (mTESE) surgery or testicular biopsy between Sep 2021 and Mar 2022 at the Center for Reproductive Medicine, Renji Hospital, Shanghai Jiaotong University School of Medicine. The study was approved by the Shanghai Jiaotong University School of Medicine, Renji Hospital Ethics Committee. Signed informed consent was obtained from each sample donor. For the diagnosis of azoospermia, semen analyses were conducted at least three times. In total, 48 patients diagnosed with NOA served as the disease group (also known as spermatogenic dysfunction group), and 12 OA patients with full spermatogenesis confirmed by histopathological examination were recruited as the control group (also known as the full spermatogenesis group). Patients with abnormal karyotypes and Y-chromosome microdeletions were excluded. These 60 samples were the testing set. The clinicopathological information of the testing set is shown in Additional file 3: Figure S1A–C. Patients’ testicular volumes or testicular lengths(L)/widths(W)/heights(H) measured by ultrasound were also collected, and the volume was calculated with formula L × W × H × 0.71 (if L/W/H data were available) [55].

### Supplementary methods

Other employed datasets [38, 56, 57] (Additional file 3: Figure S1E), methods and statistical analysis are shown in the Additional file 2: Supplementary Methods file.

## Supplementary Information

**Additional file 1: Supplementary File 1.**16 mast cell-related reference gene sets of GO collection from Molecular Signatures Database.

**Additional file 2: Supplementary Methods. **Other methods and statistical analyses of the study.

**Additional file 3: Figure S1.** Information of samples included in the study. (A) Clinicopathological information of 60 testicular samples of the testing set. CCL2 high/low groups were divided based on the median AOD value of CCL2 IHC staining. (B) Bar plot showing testicular volumes in full spermatogenesis and spermatogenic dysfunction groups of the testing set. **** p < 0.0001. (C) Scatter plots showing spearman correlations between testicular volumes (ml) and Johnsen score in the testing set. (D) Hematoxylin–eosin (HE) staining of representative samples with different pathological status in the testing set. Arrows represented testicular spermatozoa. (E) Characteristics of all testicular samples/datasets used in this study. JS, Johnsen score. Note: Only data of 58 patients with known testicular volumes from the ultrasound was used for statistical analysis in S1B-C. AOD, average optical density.

**Additional file 4: Figure S2.** Quality control metrics of scRNA-seq set. (A) Violin plots showing number of genes (left), number of UMI counts (middle) and percentage of mitochondrial genes (right) of all cell types. (B) Plot of number of genes (features) versus number of UMI counts originating from 8 samples. (C) Plot of percentage of mitochondrial genes versus number of UMI counts originating from 8 samples.

**Additional file 5: Figure S3.** Feature plots of germ cells’, somatic cells’ and immune cells’ markers in scRNA-seq set. (A)-(C) Feature plots of DDX4, VIM and PTPRC, respectively, in scRNA-seq set. (D)-(L) Feature plots of expression patterns of markers for different germ cells (SSC, diff_SPG, SPC, spermatids). Genes reflected more differentiated spermatogenic cells as they move from plot D to plot L. SSC, Spermatogonal stem cells; diff_SPG, differentiating spermatogonia; SPC, spermatocyte.

**Additional file 6: Figure S4.** IHC stained sections from HPA of germ cells’, somatic cells’ and immune cells’ markers in the testis. (A)-(C) Immunohistochemical staining of DDX4, VIM and PTPRC, respectively, in testis. Arrows represented positive cells. (D)-(I) Immunohistochemical staining of expression patterns of additional markers for different germ cells. Genes reflected more differentiated spermatogenic cells as they move from plot D to plot I. The original images of IHC stained sections were obtained from Human Protein Atlas database (https://www.proteinatlas.org/).

**Additional file 7: Figure S5.** UMAP plots of the integrated data. (A) UMAP plots for control (normal spermatogenesis) group and for disease (spermatogenic dysfunction) group were in left and right part, respectively. (B) UMAP plots of 5 control samples. (C) UMAP plots of 3 disease samples.

**Additional file 8: Figure S6.** Identification and functional enrichment of key modules correlated to both testicular mast cell infiltration and spermatogenesis using WGCNA (discovery set). (A) Sample clustering along with clinical traits. For continuous variables, color intensity changed positively with mast cell/macrophage infiltration levels or Johnsen scores. For spermatogenic dysfunction, red referred to “with spermatogenic dysfunction” wile white meant no spermatogenic dysfunction. (B) (left) Analysis of scale-free fit index and (right) mean connectivity for detecting soft-threshold power. (C) Dendrogram of all genes clustered by TOM-based dissimilarity. (D) Heatmap reflecting the relationship between module eigengenes and clinical traits. Correlation coefficient and p value were in each box. (E) Gene significance and errors among all modules associated with mast cells trait. (F) Scatter plot of module eigengenes in the blue module. (G) Bubble plot showing BP, CC and MF terms for genes in the blue module. (H) Scatter plot of module eigengenes in the turquoise module. (I) Bubble plot showing BP and CC terms for genes in the turquoise module (MF terms not enriched). WGCNA, weighted gene co-expression network analysis. TOM, topological overlap matrix. BP, Biological Process. CC, Cellular Component. MF, Molecular Function.

**Additional file 9: Figure S7.** Identification and functional annotations of DEGs in the discovery set. (A) Volcano plot showing DEGs of the discovery set. Spots in black represented not significantly differentially expressed. Spots in red were upregulated genes with “log2 fold change > 1.0 and adjusted p < 0.05” (light red) or “log2 fold change > 2.5 and adjusted p < 0.0001” (dark red). Spots in blue were downregulated genes “log2 fold change < -1.0 and adjusted p < 0.05” (light blue) or “log2 fold change < -2.5 and adjusted p < 0.0001” (dark blue). (B) Circos plot of chromosomal positions and expression profile of top 100 DEGs. The outer circle showed chromosomes, and each gene symbol pointed to its specific chromosomal location with a line. Samples from the discovery set were visualized in the inner circular heatmaps. Samples with decreasing Johnsen scores were represented from the inside (JS = 10) to the outside circles (JS = 2). The blue bar charts in the inner layer reflected -log_10_(adjust p) of each DEG. Red color represented up-regulation while blue showed downregulation. The top 5 up and down-regulated genes according to |log2 fold change| (showed in red and blue, respectively) are linked with red or blue lines in core of the plot and their IHC stained sections in the testis from HPA dataset (except for COX7A2L) were shown close to gene symbols. Note: only 97 DEGs remained after matching the 100 DEGs with the reference list. (C), (D), (E) and (F) GO-BP analysis (circular dendrogram), GO-CC, GO-MF analysis (two chord plots) and KEGG pathways (gene-concept network plot), respectively, in downregulated DEGs. (G), (H) and (I) GO-BP analysis (circular dendrogram), GO-CC analysis (chord plot) and KEGG pathways (gene-concept network plot), respectively, in upregulated DEGs. Note: MF of GO analysis was not enriched in upregulated DEGs. DEGs, differentially expressed genes. GO, gene ontology. KEGG, kyoto encyclopedia of genes and genomes.

**Additional file 10: Figure S8.** Internal (discovery set) and external (validation set 1&2) validation of 10 hub immune genes. (A), (D) and (G) Correlation heatmaps of 10 hub immune genes in the discovery set, validation set 1 and validation set 2, respectively. Pie charts showed the proportion of |spearman correlation coefficient| in 1. Blue represented positive correlation while red indicated negative correlation. Color intensity enhanced with the correlation coefficient increasing. A cross on pie chart meant the correlation of the two parameters was not significant (p > 0.05). (B), (E) and (H) Lollipop charts showing spearman correlations of 10 hub immune genes with mast cell infiltration level in the discovery set, validation set 1 and validation set 2, respectively. (C), (F) and (I) Lollipop charts showing spearman correlations of 10 hub immune genes with Johnsen scores in the discovery set, validation set 1 and validation set 2, respectively. Note: To be rigorous, for validation set 2, only samples from spermatogenic dysfunction group with clear JS marked in the original research were analyzed in Figure S8I. For lollipop charts, the length of sticks and the diameter of spots represented absolute value of correlation coefficient (Abscorr). The color of spots varied with p values of correlation analysis.

**Additional file 11: Figure S9.** Expression patterns of selected hub immune genes that show good performances in both internal and external validations. (A)-(C) Violin plot of IL13, SHC1 and IL18 (respectively) expressed in control versus disease groups. Large yellow dots indicated mean expression value, **** adjust p < 0.0001 wilcoxon rank sum test with bonferroni correction using Findmarkers function. (D) Violin plot of IL13 expressed in different testicular cell types. (E) and (F) Immunohistochemical staining of SHC1 and IL18, respectively, in the testis. The original images of IHC stained sections were obtained from Human Protein Atlas database (https://www.proteinatlas.org/). Note that there’s no IL13 IHC staining data of the testis in HPA.

**Additional file 12: Figure S10.** Scatter plots with loess fitting curves showing the relationship between CCL2 expression and mast cell infiltration (left column) or JS (right column). (A), (C) and (E) Scatter plots showing the spearman correlations between CCL2 expression level and mast cell infiltration level in the discovery set (A), validation set 1 (C) and validation set 2 (E). (B), (D) and (F) Scatter plots showing the spearman correlations between CCL2 expression level and Johnsen scores (or modified Johnsen scores) in the discovery set (B), validation set 1 (D) and validation set 2 (F). Note: To be rigorous, for validation set 2, only samples from spermatogenic dysfunction group with clear JS marked in the original research were analyzed in S10F.

**Additional file 13: Figure S11.** Heatmaps of scores from GSVA analyses on 16 mast cell related signatures in the discovery set (A), validation set 1 (B) and validation set 2 (C).

**Additional file 14: Figure S12.** Potential role of endothelial cells (ECs) in mast cell chemotaxis. (A) Bar plot showing log2 fold change (logFC) of mast-cell related pathways GSVA scores originated from ECs (logFC were obtained by comparing scores from ECs in disease group versus control group). Blue color indicated enhancement in the ECs of disease group. Dark blue meant between-group adjust p < 0.05 (BH adjustment using the limma package). (B) Immunohistochemical staining of SELE in one testis. Arrows represented potential positive cells. The original image of SELE IHC stained section was obtained from Human Protein Atlas database (https://www.proteinatlas.org). (C) Violin plots showing expression level of SELE among different testicular cell types in testes. The violin plots were split by groups. (D)-(E) Circle plots reflecting SELE signal pathway networks in control (D) and disease (E) groups. (F) Bubble plot of detailed SELE signal pathways in cell–cell chats of testes.

**Additional file 15: Table S1.** ssGSEA scores of 3 main immune cell types of testes based on 24 immune cells signatures from Bindea et al.

**Additional file 16: Table S2.** Gene lists of all modules identified by WGCNA.

**Additional file 17: Table S3.** DEGs between patients with normal spermatogenesis and spermatogenic dysfunction in the discovery set.

**Additional file 18: Table S4.** 111 intersected genes from immune genes ∩ DEGs ∩ blue/turquoise module genes.

**Additional file 19: Table S5.** GSEA results based on KEGG set of spermatogenic dysfunction patients with high versus low CCL2 expression in the discovery and validation sets.

## Data Availability

The raw ScRNA-seq and microarray data used in the current study are available at GEO or Arrayexpress database with corresponding reference numbers listed in Additional file 3: Figure S1E. All generated or analyzed data of this study are included in this published article and its supplementary files. The raw data of patients/samples and in vitro experiments from our own institutes are available from corresponding authors on reasonable request.
