# Supplementary material for: Identification and validation of CCL2 as a potential biomarker relevant to mast cell infiltration in the testicular immune microenvironment of spermatogenic dysfunction
Source: Cell Biosci. 2023 May 23;13:94. doi: 10.1186/s13578-023-01034-2 (PMC10204296; doi:10.1186/s13578-023-01034-2)
Supplement: Supplementary file 2 — Additional file 2: Supplementary Methods. Other methods and statistical analyses of the study. [file 13578_2023_1034_MOESM2_ESM.pdf]

Supplementary Methods

Identification and validation of CCL2 as a potential biomarker relevant to mast cell infiltration in the testicular immune microenvironment of spermatogenic dysfunction

Fan Dong[1,2], Ping Ping[1,2], Si-Qi Wang[1,2], Yi Ma[1,2#], Xiang-Feng Chen[1,2,3#]

1. Center for Reproductive Medicine, Ren Ji Hospital, School of Medicine, Shanghai Jiao Tong University, Shanghai, China.
2. Shanghai Key Laboratory for Assisted Reproduction and Reproductive Genetics, Shanghai, China.
3. Shanghai Human Sperm Bank, Shanghai China.

#Corresponding to Dr. Xiang-Feng Chen & Dr. Yi Ma

Histological examination and Immunohistochemical staining

Samples were fixed in Bouin's fixative (Phygene Biotech,#LA0976), embedded in paraffin and sectioned into 4 μm thin for immunohistochemical (IHC) and hematoxylin-eosin (HE) staining. IHC staining was based on a three-step method. In brief, the embedded testicular sections were deparaffinized in dimethylbenzene and then rehydrated in graded ethanol (100%,100% 85% and 75%) followed by distilled water wash. Tris-EDTA (PH=9.0) or citrate antigen retrieval buffer (PH=6.0, Servicebio, #G1202, for Tryptase IHC staining) was used for antigen retrieval and the sections were then washed by PBS for 3 times. 3%H_2O_2 was used to block endogenous peroxidase in room temperature for 25 min, followed by 3 times PBS wash. 3% bovine serum albumin (BSA) was used to block non-specific binding sites for 30 minutes. Next, the sections were incubated with primary antibodies including anti-CCL2(1:200, Affinity, #BF0556) and anti-Tryptase (1:200000,Abcam, #AB2378) at 4℃ overnight, followed by 3 times PBS wash. HRP conjugated goat anti-mouse IgG (1:200, Servicebio, #GB23301) was used as second antibody in room temperature for 50 minutes. DAB chromogen solution (DAKO, #K5007) was dropped onto the tissue to recognize positive reaction. Hematoxylin solution (Servicebio, #G1004) was employed for nuclear staining. Afterwards, the sections were dehydrated in graded ethanol (75%, 85%, 100% and 100%) and hyalinized in dimethylbenzene. IHC negative control was prepared by omitting the corresponding primary antibodies. The sealed slides of testicular sections were visualized using microscope (Olympus, #CX43) and were digitalized using digital pathology slide scanner (KFBIO, #KF-PRO-120). Besides, for some other important markers or genes, the IHC stained sections were downloaded from Human Protein Atlas database [1] (https://www.proteinatlas.org). For histopathological examination, the rehydrated sections were stained with Hematoxylin-Eosin (HE) and were similarly dehydrated and hyalinized. The histopathological examination of the HE stained sections for each patient was conducted by two independent observers and JS were determined for each sample [2]. Representative samples with different histopathological status were shown in Additional file 3: Figure S1D.

Immunofluorescence staining

Maybe due to the acidic characteristic of bouin's fixative, we found that the nuclei of testicular samples fixed in Bouin's fixative were difficult to get stained by DAPI dye. The 4% paraformaldehyde fixed testicular section, although could be stained with DAPI dye, might have changed morphology [3]. To solve this dilemma, another three testicular samples (one control and two diseases) were fixed in animal testicular tissue fixative (Servicebio, #G1121), which is a special fixative for testicular tissue, and were specially used for immunofluorescence (IF) staining. Sections were also stained with HE and were histopathological confirmed. For IF staining, after deparaffinization and rehydration, antigen was retrieved with Tris-EDTA (PH=8.0) and non-specific sites were blocked as described in IHC staining. Next, the sections were incubated at 4℃ overnight with anti-CCL2(mouse,1:500, Affinity, #BF0556) together with one of other rabbit antibodies, including anti-α-SMA (1:200, Affinity, #AF1032), and STAR (1:100,Abclonal,#A16432). The sections were washed by PBS and then incubated with second antibodies including Cy3 conjugated Goat anti-mouse IgG (1:300, Servicebio, #GB21301) and Alexa Fluor 488-conjugated Goat anti-rabbit IgG (1:400, Servicebio, #GB25303). Then autofluo quencher (Servicebio, #G1221) was added to sections for 5 min

and washed in order to decline the autofluorescence of the tissue. Afterwards, DAPI dye (Servicebio, #G1012) was used for nuclear staining. The slides were sealed with anti-fluorescence quenching mounting agent (Servicebio, #G1401), visualized under fluorescence microscope (NIKON, Eclipse C1) and digitalized by fluorescent slide scanner (Pannoramic MIDI,3DHISTECH).

Cell culture and reagent treatment

Mouse spermatogonia cell line GC-1 (ATCC, VA, USA) and spermatocyte cell line GC-2 (Chinese Academy of Sciences Cell Bank, Shanghai, CN) were cultured in DMEM (Gibco,#11995-065) with 10% fetal bovine serum (VivaCell, #C04001-500) and 1% penicillin-streptomycin solution (Gibco, #15140-122) at 37℃ with 5% CO_2. The medium was replaced every two days. Recombinant mouse Ccl2 (Novoprotein, #CR09) was dissolved in double distilled water (ddH_2O) and Ccl2 solution was used to investigate the effect of Ccl2 on germ cells in vitro. The concentration of Ccl2 treatment was set as 100ng/ml according to previous report [4].

Colony formation assay

GC-1 and GC-2 Cells were seeded into 6-well plates (around 300 cells per well) at day1 and added with Ccl2 solution (Ccl2 group) or ddH_2O (control group). Cells were next cultured for seven days to form clones. At day8, the medium was removed and cells were washed with PBS for three times, fixed with 4% paraformaldehyde for 20min and stained with 1% crystal violet (#G1062, Solarbio) for 20min. The plates were then washed by running water and photographed after drying. The experiments were repeated three times.

Microarray data collecting and processing

The microarray data used in this study were listed in Additional file 3: Figure S1E. For GSE4797, the raw TXT files from CodeLink microarray platform were downloaded from Gene Expression Omnibus (GEO) dataset and were loaded with codelink package (v1.60.0) [5]. GPL2891 annotation file was downloaded from https://www.ncbi.nlm.nih.gov/geo/query/acc.cgi?view=data&acc=GPL2891&id=12727&db=GeoDb_blob06. As GSE4797 raw data were with legacy probe names, instead of current ones, and in some samples the Feature_id column was missing, we used a workaround (listed in https://support.bioconductor.org/p/68532/#68849) offered by Dr.Diego Diez, the author and maintainer of the codelink package, to deal with raw data. Briefly, the raw ".TXT" files were loaded with readCodelinkSet function, conducting RMA correction (parameters set: method = "normexp", normexp.method="rma"), and quantile normalization (parameters set: method="quantile"). Thereafter, the above-mentioned GPL file was used to match "LOGICAL_COL/LOGICAL_ROW" columns of the original data with the Feature_id and currently used probe names (listed in the GPL file). Then, the probe-matrix with currently used probe names as rownames and sample ids as colnames was annotated with gene symbol using h20kcodSYMBOL function of h20kcod.db package (v3.4.0). For genes with missing value in more than 20% samples, the rows were deleted. The rest missing value were supplemented by k-Nearest Neighbor algorithm [6] and average expression were obtained for duplicate genes. Afterwards, the expression gene matrix was renormalized using

normalizeBetweenArrays function of the limma package (v3.48.1) before using. For GSE45885, the raw ".cel" files were loaded with the oligo package (v1.56.0). The probe-matrix was then corrected and normalized using rma function and hugene10sttranscriptcluster.db (v8.8.0) was used for converting probe names to gene symbols. Average expression was used for duplicate genes and the gene-matrix were handled with normalizeBetweenArrays before analyzing. For E-TABM-1214, the raw ".cel" data of all 47 samples were downloaded from Arrayexpress dataset [7] and were together loaded with the affy package (v1.70.0). After being processed with rma function, hgu133plus2.db (v3.13.0) were used for probe to symbol conversion. Average expression was obtained for duplicate genes and gene expression matrix was renormalized with normalizeBetweenArrays. Then the data of 38 adult samples were further extracted out of the whole 47 samples' matrix for analyses while the juvenile samples were excluded. The JS or mJS of all samples were obtained from the corresponding original articles or datasets.

Immune cells infiltration analysis of microarray samples

The single sample gene set enrichment analysis (ssGSEA) algorithm [8] was carried out with the GSVA package (v1.40.1) to quantify the abundance of immune cells infiltrating in the testicular microenvironment, based on widely-used gene sets of Bindea 24 immune cells signature [9-15] acquired from previously published article [9], which was originated from the work of Bindea et al [16].

WGCNA and key modules identification

The top 5000 genes selected by median absolute deviation (MAD) of discovery set were extracted to construct WGCNA [17] using the WGCNA package (1.70-3). The soft-threshold power was set as 7 (scale-free R2= 0.91, slope=-1.19). Then the adjacency matrix was converted to a topological overlap matrix (TOM) and the dissTOM (1-TOM) matrix was calculated. Genes clustering tree was drawn with hclust function based on TOM-based dissimilarity. Thereafter, dynamic hybrid cutting method were employed to create the clustering dendrogram with "minModuleSize" set as 30. Then the similar modules were clustered and merged with "MEDissThres" set as 0.25. Pearson correlations between module eigengenes and selected clinical traits were calculated. And modules with high correlation with both testicular mast cell infiltration level and spermatogenic function were considered as key modules.

Function enrichment analyses of genes in key modules

The genes of key modules were extracted and used for functional enrichment analysis using the clusterProfiler package [18, 19]. Gene Ontology (GO) terms were set as a reference and pathways with adjusted p<0.05 were defined as significantly enriched pathways.

DEGs identification and function enrichment analyses of microarray data

DEGs between full spermatogenesis and spermatogenic dysfunction groups of the discovery set were identified by the limma package (v3.48.1) [20]. Genes with adjusted p value (using false discovery rate method) <0.05 and |log2 fold change|(logFC) >1 were defined as DEGs. A volcano plot was drawn to show all the DEGs. The expression characteristics of the top 100

DEGs (top 50 up-regulated and top 50 down-regulated according to |logFC|) and their location on chromosome were visualized using the OmicCircos package (v1.30.0). All DEGs were extracted to complete GO and Kyoto Encyclopedia of Genes and Genomes (KEGG) pathways analyses using the above-mentioned methods.

Acquisition of immune genes list and intersection of key modules/DEGs/immune genes list

Immunological genes list was obtained from the immunology database and analysis portal (ImmPort) dataset [21] (https://s3.immport.org/release/genelists/GeneList.txt?download=true, Updated: July 2020). The duplicate gene symbols were removed and a total of 1793 deduplicated immune-related genes were acquired and was defined as the immune gene list of our study. The intersections of immune gene list, DEGs list, and gene list of key modules were obtained and Venn diagrams were drawn to show the intersected genes using the VennDiagram package (v1.6.20).

Identification of hub immune genes related to both testicular mast cell infiltration and spermatogenic function

Intersected gene list was input into Search Tool for the Retrieval of Interacting Genes （STRING） database [22] to construct a protein-protein interaction (PPI) network (parameters set: minimum required interaction score=0.7, hide disconnected nodes =True). The PPI network was downloaded and then further analyzed by Cytoscape (v 3.7.2). The maximum clique centrality (MCC) algorithm of CytoHubba plug-in [23] was used to find hub genes. The top 10 genes with highest MCC values were identified as hub immune genes, which were the key immune-related genes that were relevant to both testicular mast cell infiltration and spermatogenic function.

Internal and external validation and further filtration of hub immune genes

The discovery set itself was used to do hub genes' internal validation. Two validation sets were employed to externally validate and further filter these hub immune genes from two angles. First, the spearman correlations between the expression of hub immune genes and mast cell infiltration level were worked out using cor function and ggcorrplot package (v0.1.3), and the results were summarized by ggdotchart function of ggpub package (v0.4.0). Second, the spearman correlations between the expression of hub immune genes and JS (or mJS) were calculated (also summarized by ggdotchart) in order to validate the relationship between hub genes and spermatogenic function. Correlation heatmaps based on 10 hub genes were constructed by corrplot (v0.90). The interested hub gene was picked out for further study according to their performance in the validation and their functions reported by previous literatures. For the individual interested hub gene, its correlations with mast cell infiltration or JS in the discovery set and validation sets were illustrated by packages ggplot2 (v3.3.5). Next, the expression of interested hub gene was validated in the testing set. And spearman correlations between its expression and mast cell infiltration level, testicular volumes and JS were also validated in the testing set. Correlations between the expression of the interested hub gene and mast cell infiltration level/testicular volume in the testing set were visualized by ggscatterstats function of ggstatsplot (v0.9.3). Receiver operating characteristic (ROC) curves

of the interested hub gene were plotted in GraphPad Prism (v9.2.0).

Gene set variation analysis (GSVA) and gene set enrichment analysis (GSEA) of microarray data

GSVA [24] was carried out using the GSVA package. The 16 reference gene sets related to mast cells (Additional file 1: Supplementary File 1) were obtained from GO collection and was downloaded from Molecular Signatures Database (MSigDB) (http://www.gsea-msigdb.org/gsea/msigdb/index.jsp). Thereafter, the GSVA results were visualized using pheatmap (v1.0.12), and the correlations between GSVA scores of each sample and the expression level of interested hub gene were calculated. Moreover, GSEA [25] was conducted to further evaluate the molecular mechanisms of a certain interested hub gene involved in spermatogenic dysfunction. First, the samples were divided into two clusters according to the median expression value of an interested hub gene to be studied (high versus low expression clusters). Second, differential expression analysis was conducted between the two clusters and a logFC value was get for each gene. Third, all genes were ranked by logFC, and the ranked gene list was subjected to GSEA, which was conducted by clusterProfiler (18, 19) （parameters set: exponent = 1, minGSSize = 10, maxGSSize = 500, by = "fgsea") based on "c2.cp.kegg.v7.4.entrez.gmt" set (downloaded from MSigDB).

Construction of interested hub gene-related immune PPI network in testis

We constructed a testicular immune PPI network related to the interested hub gene. First, the immune gene list was used to separately get its intersections with genes of the discovery set, validation set 1 and 2 (so we get three immune sub-lists). Next the intersection of those three immune sub-lists was obtained, which referred to the commonly expressed immune genes in the testis. Third, spearman correlations between the expression level of interested hub gene and commonly expressed immune genes were calculated in discovery and validation sets, and genes that maintained all rho>0.5 along with p<0.05 among all three datasets were considered as interested hub gene-related testicular immune genes. These genes (along with the interested hub gene) were input into STRING database to construct a hub gene-related testicular immune PPI network (parameters set: minimum required interaction score=0.7, hide disconnected nodes =True). And the network was downloaded and processed by Cytoscape (the immune proteins not connected to the main network were removed). Therefore, the interested hub gene-related testicular immune PPI network was established and immune proteins were categorized according to gene functions (the categories were simplified according to the "Category" column of the original immune gene list from ImmPort).

Statistical analysis

Data were shown as mean±SD. For group comparison, Mann–Whitney test (two groups) or Kruskal–Wallis test (three or more groups) and Dunn multiple-comparisons for *post-hoc* examination were used. The correlations between gene expression level/GSVA score and JS/immune cell infiltration level were based on spearman correlation analyses. For semi-quantitative analysis of CCL2 in the testing set, five random microscopic high power fields (HPFs) (40×) of each IHC staining image were captured. The Integrated Optical Density (IOD) and Tissue Quantification Area (Area, calculated by pixel) of each field were determined with Image Pro Plus 6.0 (v6.0.0.260). Finally, we used Average Optical Density (AOD= IOD/Area)

for statistical calculation. For mast cell infiltration intensity of the testing set, we also captured five random microscopic HPFs of each slide and positive cells were quantified manually in Image Pro Plus 6.0 with Tissue Quantification Area (Area) presented as mm^2. The final infiltration level of mast cells was presented as cells/mm^2. For colony formation assay, the pictures of each well were separately analyzed using ImageJ (v1.53k) plugin ColonyArea [26], and colony intensity percentage (CIP) was used for statistical analysis. All statistical tests were performed in R studio or GraphPad Prism (v9.2.0). Statistical significance was set at p value<0.05.

References

1. Uhlen M, Fagerberg L, Hallstrom BM, Lindskog C, Oksvold P, Mardinoglu A, et al. Proteomics. Tissue-based map of the human proteome. Science. 2015;347(6220):1260419.
2. Johnsen SG. Testicular biopsy score count--a method for registration of spermatogenesis in human testes: normal values and results in 335 hypogonadal males. Hormones. 1970;1(1):2-25.
3. Tang RL, Fan LQ. PLZF(pos)c-KIT(pos)-delineated A1-A4-differentiating spermatogonia by subset and stage detection upon Bouin fixation. Asian J Androl. 2019;21(3):309-18.
4. Wang S, Qian Z, Ge X, Li C, Xue M, Liang K, et al. LncRNA Tug1 maintains blood-testis barrier integrity by modulating Ccl2 expression in high-fat diet mice. Cell Mol Life Sci. 2022;79(2):114.
5. Diez D, Alvarez R, Dopazo A. Codelink: an R package for analysis of GE healthcare gene expression bioarrays. Bioinformatics. 2007;23(9):1168-9.
6. Troyanskaya O, Cantor M, Sherlock G, Brown P, Hastie T, Tibshirani R, et al. Missing value estimation methods for DNA microarrays. Bioinformatics. 2001;17(6):520-5.
7. Parkinson H, Kapushesky M, Shojatalab M, Abeygunawardena N, Coulson R, Farne A, et al. ArrayExpress--a public database of microarray experiments and gene expression profiles. Nucleic Acids Res. 2007;35(Database issue):D747-50.
8. Barbie DA, Tamayo P, Boehm JS, Kim SY, Moody SE, Dunn IF, et al. Systematic RNA interference reveals that oncogenic KRAS-driven cancers require TBK1. Nature. 2009;462(7269):108-12.
9. Senbabaoglu Y, Gejman RS, Winer AG, Liu M, Van Allen EM, de Velasco G, et al. Tumor immune microenvironment characterization in clear cell renal cell carcinoma identifies prognostic and immunotherapeutically relevant messenger RNA signatures. Genome Biol. 2016;17(1):231.
10. Jiang Y, Zheng B, Yang Y, Li X, Han J. Identification of Somatic Mutation-Driven Immune Cells by Integrating Genomic and Transcriptome Data. Front Cell Dev Biol. 2021;9:715275.
11. Thorsson V, Gibbs DL, Brown SD, Wolf D, Bortone DS, Ou Yang TH, et al. The Immune Landscape of Cancer. Immunity. 2018;48(4):812-30 e14.
12. van der Heijden M, Essers PBM, de Jong MC, de Roest RH, Sanduleanu S, Verhagen CVM, et al. Biological Determinants of Chemo-Radiotherapy Response in HPV-Negative Head and Neck Cancer: A Multicentric External Validation. Front Oncol. 2019;9:1470.
13. Zhang LY, Jin Y, Xia PH, Lin J, Ma JC, Li T, et al. Integrated analysis reveals distinct molecular, clinical, and immunological features of B7-H3 in acute myeloid leukemia. Cancer Med. 2021;10(21):7831-46.
14. Sanchez A, Furberg H, Kuo F, Vuong L, Ged Y, Patil S, et al. Transcriptomic signatures related to the obesity paradox in patients with clear cell renal cell carcinoma: a cohort study. Lancet Oncol. 2020;21(2):283-93.

15. Hakimi AA, Voss MH, Kuo F, Sanchez A, Liu M, Nixon BG, et al. Transcriptomic Profiling of the Tumor Microenvironment Reveals Distinct Subgroups of Clear Cell Renal Cell Cancer: Data from a Randomized Phase III Trial. Cancer Discov. 2019;9(4):510-25.
16. Bindea G, Mlecnik B, Tosolini M, Kirilovsky A, Waldner M, Obenauf AC, et al. Spatiotemporal dynamics of intratumoral immune cells reveal the immune landscape in human cancer. Immunity. 2013;39(4):782-95.
17. Langfelder P, Horvath S. WGCNA: an R package for weighted correlation network analysis. BMC Bioinformatics. 2008;9:559.
18. Wu T, Hu E, Xu S, Chen M, Guo P, Dai Z, et al. clusterProfiler 4.0: A universal enrichment tool for interpreting omics data. Innovation (N Y). 2021;2(3):100141.
19. Yu G, Wang LG, Han Y, He QY. clusterProfiler: an R package for comparing biological themes among gene clusters. OMICS. 2012;16(5):284-7.
20. Ritchie ME, Phipson B, Wu D, Hu Y, Law CW, Shi W, et al. limma powers differential expression analyses for RNA-sequencing and microarray studies. Nucleic Acids Res. 2015;43(7):e47.
21. Bhattacharya S, Dunn P, Thomas CG, Smith B, Schaefer H, Chen J, et al. ImmPort, toward repurposing of open access immunological assay data for translational and clinical research. Sci Data. 2018;5:180015.
22. Szklarczyk D, Gable AL, Nastou KC, Lyon D, Kirsch R, Pyysalo S, et al. The STRING database in 2021: customizable protein-protein networks, and functional characterization of user-uploaded gene/measurement sets. Nucleic Acids Res. 2021;49(D1):D605-D12.
23. Chin CH, Chen SH, Wu HH, Ho CW, Ko MT, Lin CY. cytoHubba: identifying hub objects and sub-networks from complex interactome. BMC Syst Biol. 2014;8 Suppl 4:S11.
24. Hanzelmann S, Castelo R, Guinney J. GSVA: gene set variation analysis for microarray and RNA-seq data. BMC Bioinformatics. 2013;14:7.
25. Subramanian A, Tamayo P, Mootha VK, Mukherjee S, Ebert BL, Gillette MA, et al. Gene set enrichment analysis: a knowledge-based approach for interpreting genome-wide expression profiles. Proc Natl Acad Sci U S A. 2005;102(43):15545-50.
26. Guzman C, Bagga M, Kaur A, Westermarck J, Abankwa D. ColonyArea: an ImageJ plugin to automatically quantify colony formation in clonogenic assays. PLoS One. 2014;9(3):e92444.
