# Supplementary figures and images for: Identification and validation of CCL2 as a potential biomarker relevant to mast cell infiltration in the testicular immune microenvironment of spermatogenic dysfunction
Source: Cell Biosci. 2023 May 23;13:94. doi: 10.1186/s13578-023-01034-2 (PMC10204296; doi:10.1186/s13578-023-01034-2)

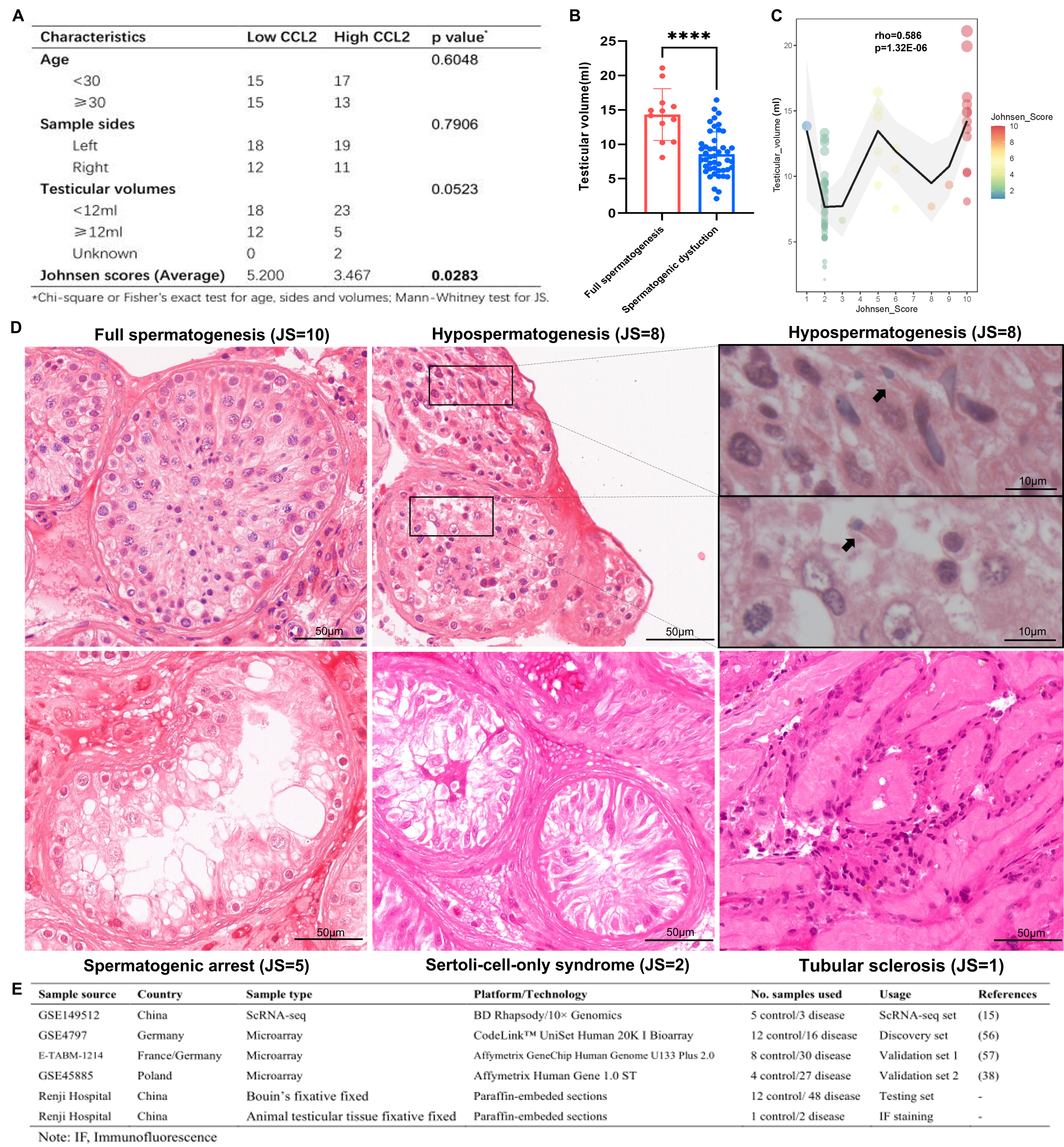

Supplement: Supplementary file 3 — Additional file 3: Figure S1. Information of samples included in the study. (A) Clinicopathological information of 60 testicular samples of the testing set. CCL2 high/low groups were divided based on the median AOD value of CCL2 IHC staining. (B) Bar plot showing testicular volumes in full spermatogenesis and spermatogenic dysfunction groups of the testing set. **** p < 0.0001. (C) Scatter plots showing spearman correlations between testicular volumes (ml) and Johnsen score in the testing set. (D) Hematoxylin–eosin (HE) staining of representative samples with different pathological status in the testing set. Arrows represented testicular spermatozoa. (E) Characteristics of all testicular samples/datasets used in this study. JS, Johnsen score. Note: Only data of 58 patients with known testicular volumes from the ultrasound was used for statistical analysis in S1B-C. AOD, average optical density. [file 13578_2023_1034_MOESM3_ESM.tif]

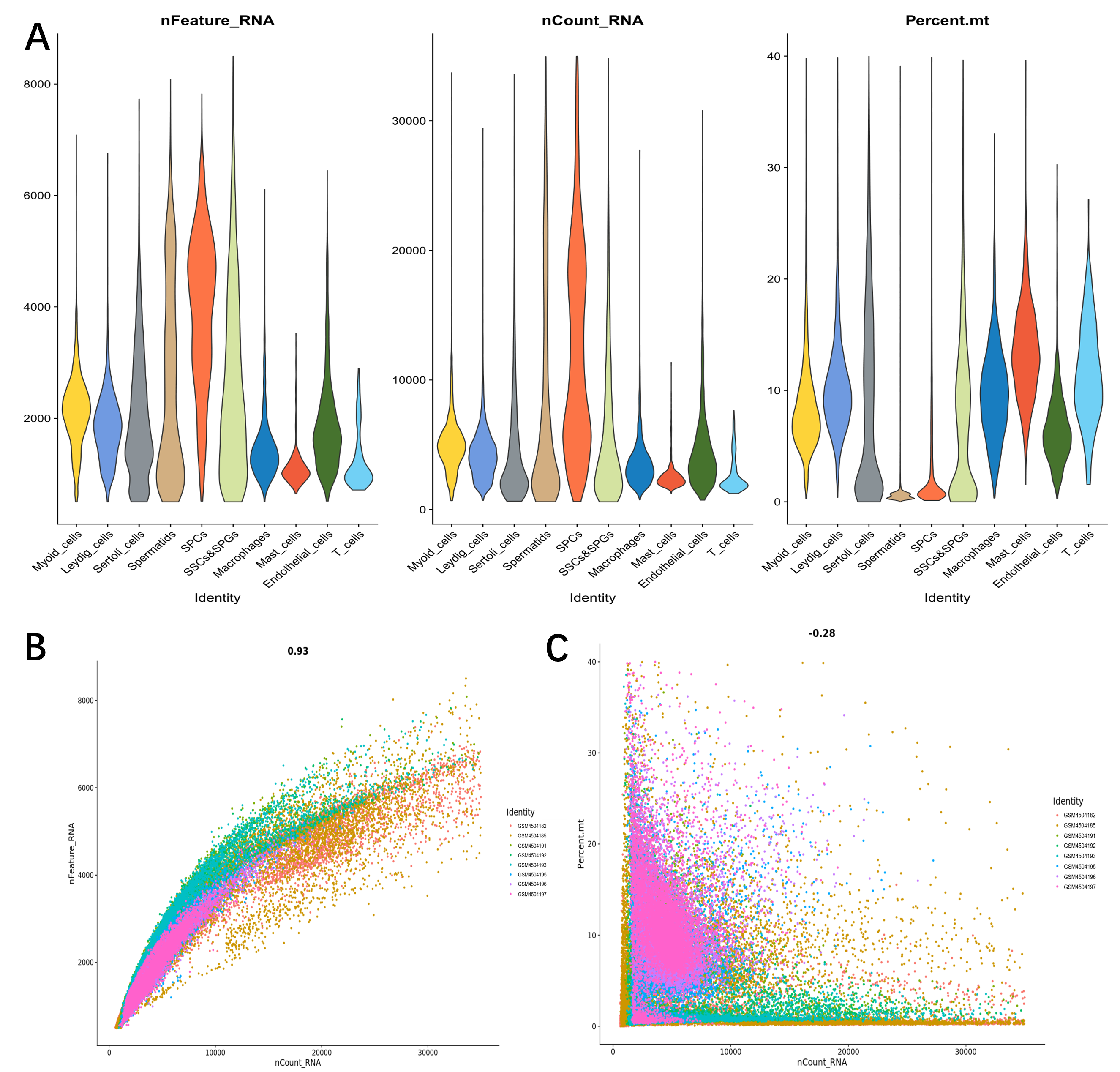

Supplement: Supplementary file 4 — Additional file 4: Figure S2. Quality control metrics of scRNA-seq set. (A) Violin plots showing number of genes (left), number of UMI counts (middle) and percentage of mitochondrial genes (right) of all cell types. (B) Plot of number of genes (features) versus number of UMI counts originating from 8 samples. (C) Plot of percentage of mitochondrial genes versus number of UMI counts originating from 8 samples. [file 13578_2023_1034_MOESM4_ESM.tif]

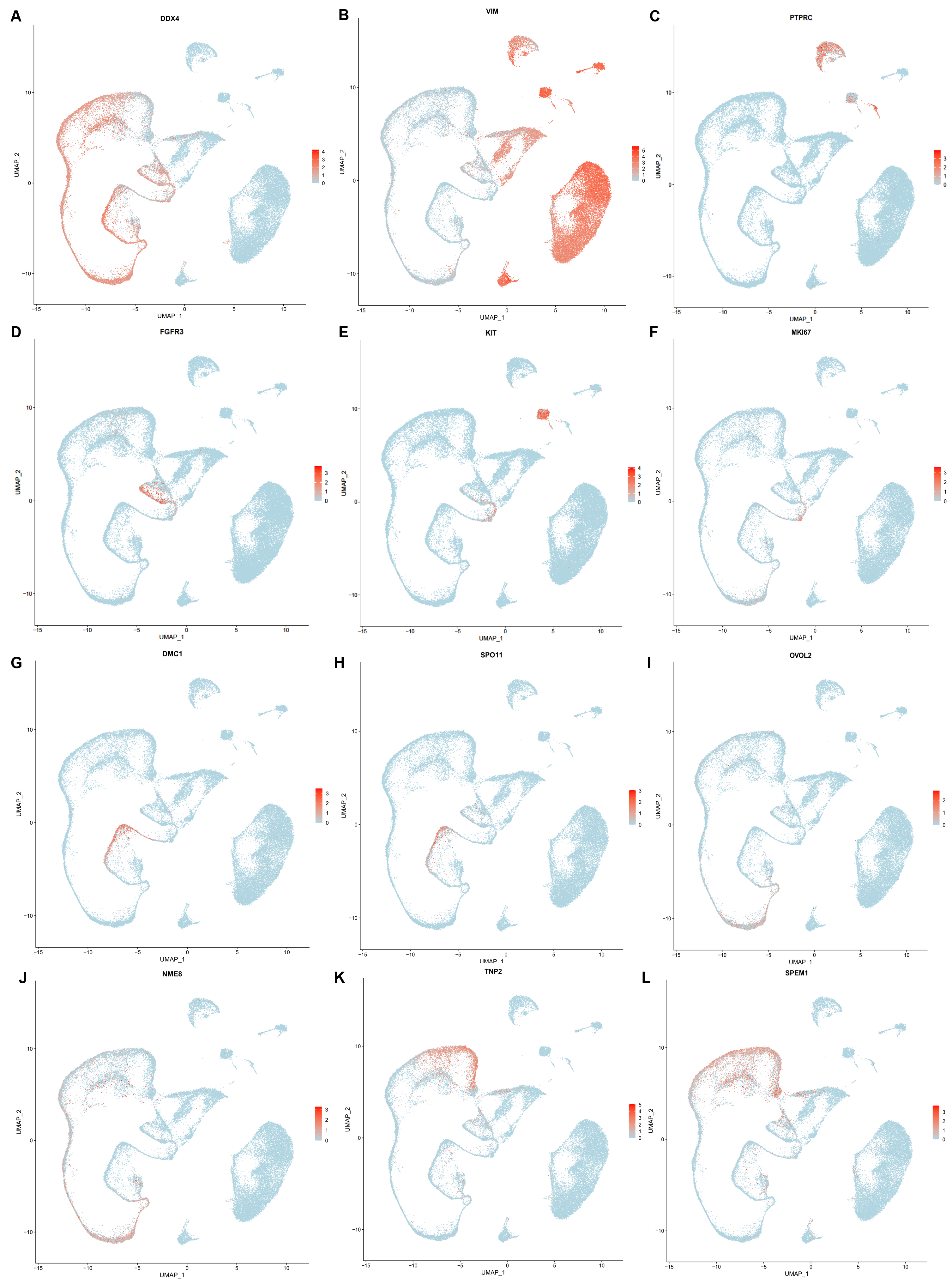

Supplement: Supplementary file 5 — Additional file 5: Figure S3. Feature plots of germ cells’, somatic cells’ and immune cells’ markers in scRNA-seq set. (A)-(C) Feature plots of DDX4, VIM and PTPRC, respectively, in scRNA-seq set. (D)-(L) Feature plots of expression patterns of markers for different germ cells (SSC, diff_SPG, SPC, spermatids). Genes reflected more differentiated spermatogenic cells as they move from plot D to plot L. SSC, Spermatogonal stem cells; diff_SPG, differentiating spermatogonia; SPC, spermatocyte. [file 13578_2023_1034_MOESM5_ESM.tif]

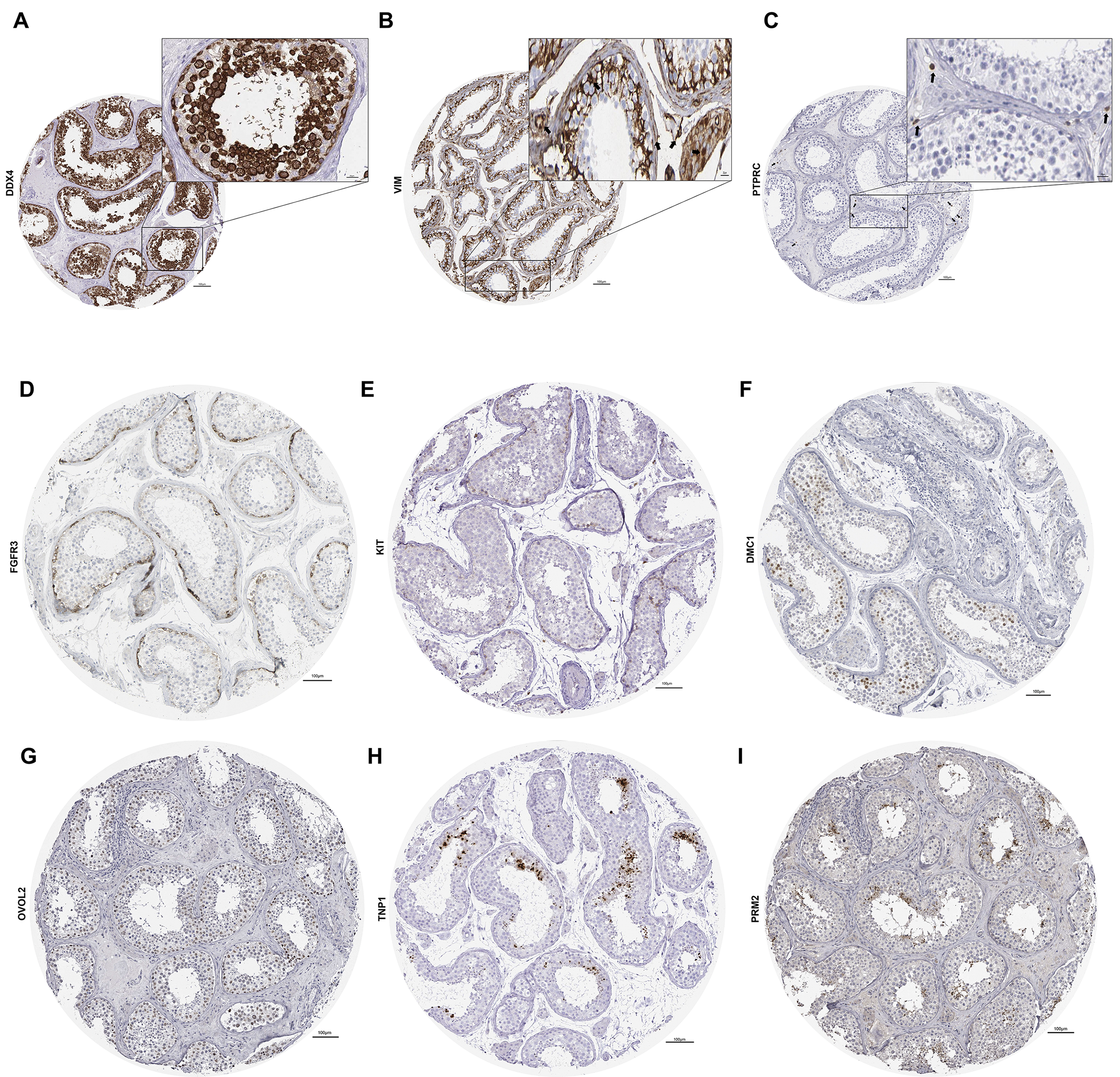

Supplement: Supplementary file 6 — Additional file 6: Figure S4. IHC stained sections from HPA of germ cells’, somatic cells’ and immune cells’ markers in the testis. (A)-(C) Immunohistochemical staining of DDX4, VIM and PTPRC, respectively, in testis. Arrows represented positive cells. (D)-(I) Immunohistochemical staining of expression patterns of additional markers for different germ cells. Genes reflected more differentiated spermatogenic cells as they move from plot D to plot I. The original images of IHC stained sections were obtained from Human Protein Atlas database (https://www.proteinatlas.org/). [file 13578_2023_1034_MOESM6_ESM.tif]

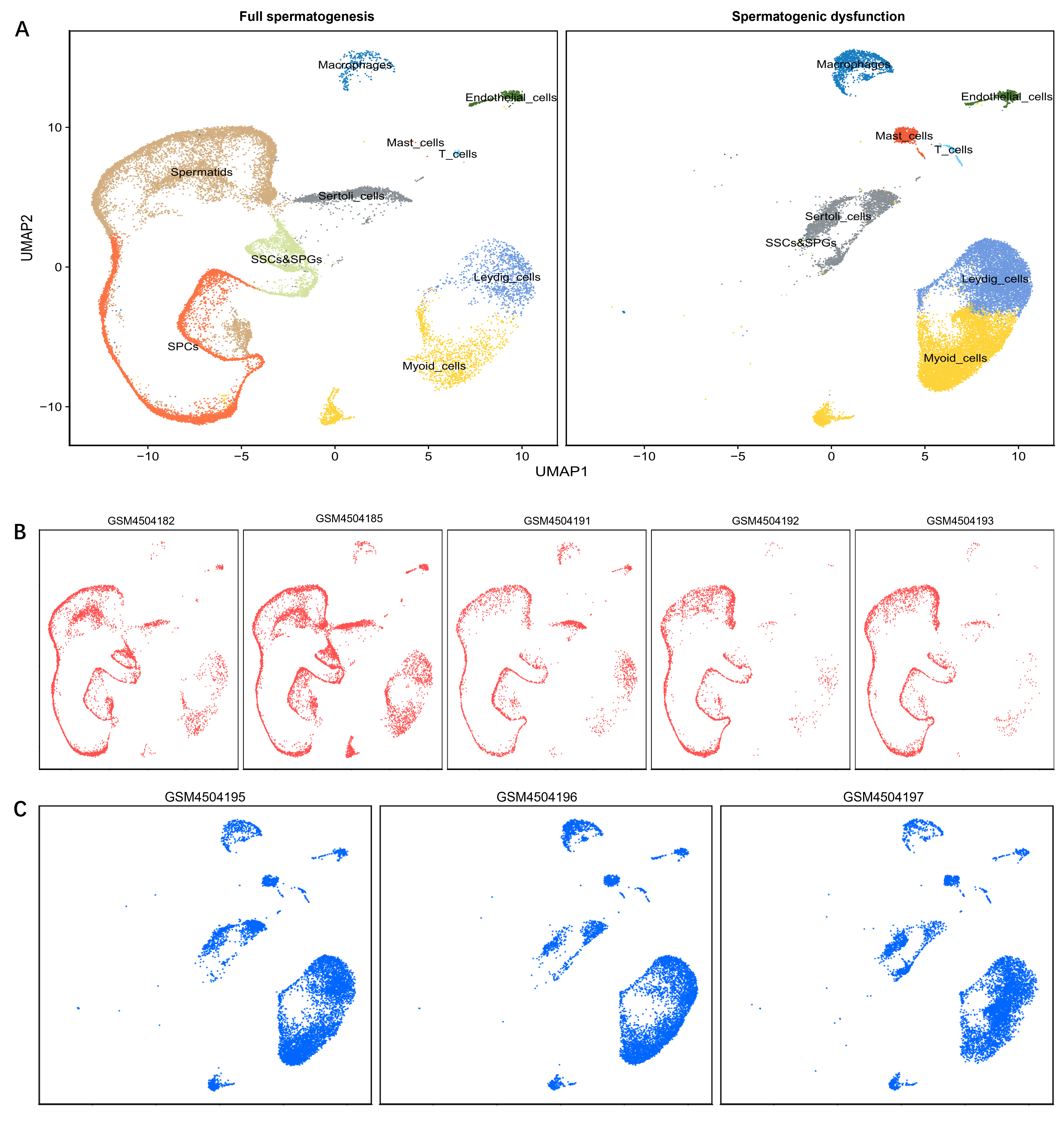

Supplement: Supplementary file 7 — Additional file 7: Figure S5. UMAP plots of the integrated data. (A) UMAP plots for control (normal spermatogenesis) group and for disease (spermatogenic dysfunction) group were in left and right part, respectively. (B) UMAP plots of 5 control samples. (C) UMAP plots of 3 disease samples. [file 13578_2023_1034_MOESM7_ESM.tif]

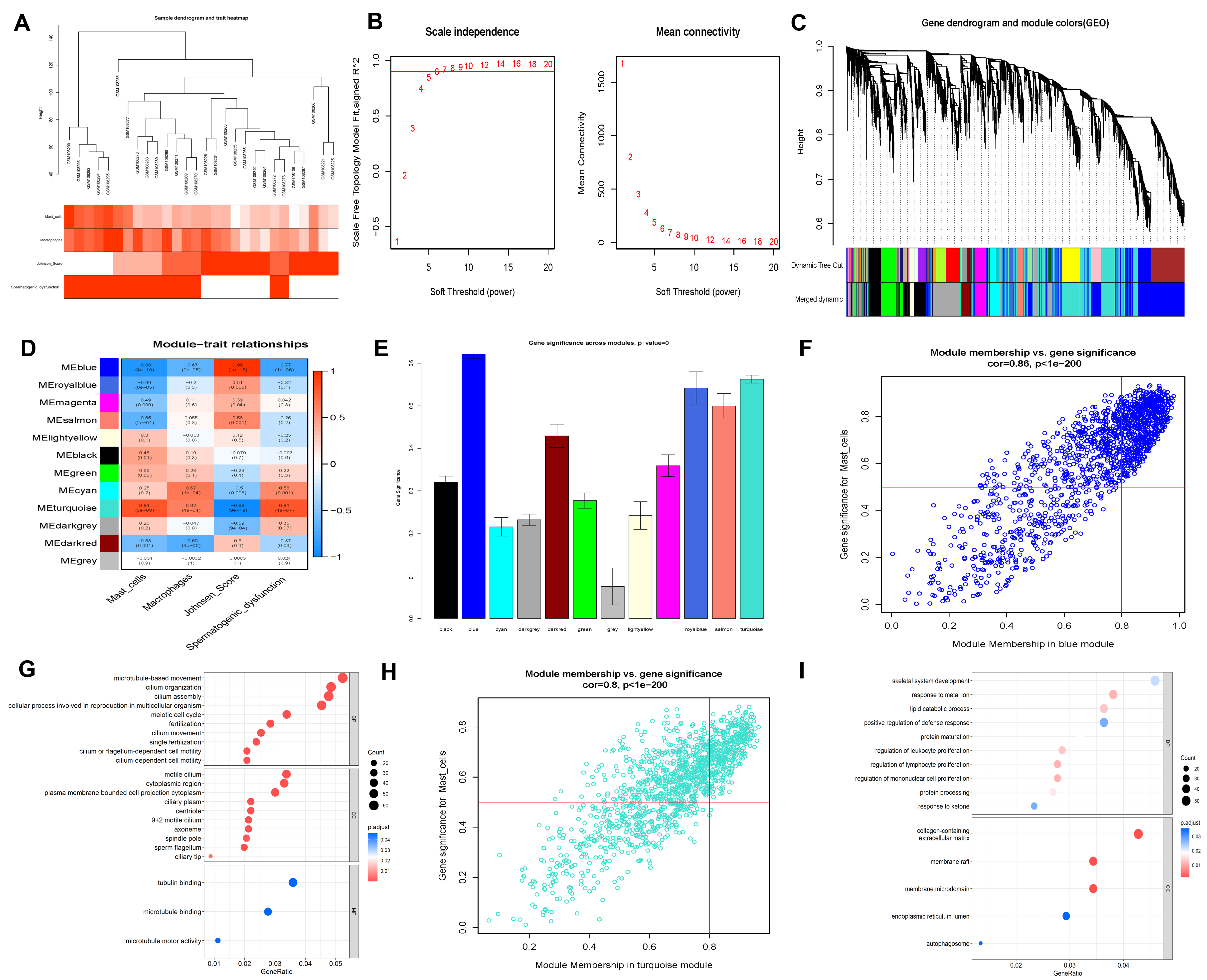

Supplement: Supplementary file 8 — Additional file 8: Figure S6. Identification and functional enrichment of key modules correlated to both testicular mast cell infiltration and spermatogenesis using WGCNA (discovery set). (A) Sample clustering along with clinical traits. For continuous variables, color intensity changed positively with mast cell/macrophage infiltration levels or Johnsen scores. For spermatogenic dysfunction, red referred to “with spermatogenic dysfunction” wile white meant no spermatogenic dysfunction. (B) (left) Analysis of scale-free fit index and (right) mean connectivity for detecting soft-threshold power. (C) Dendrogram of all genes clustered by TOM-based dissimilarity. (D) Heatmap reflecting the relationship between module eigengenes and clinical traits. Correlation coefficient and p value were in each box. (E) Gene significance and errors among all modules associated with mast cells trait. (F) Scatter plot of module eigengenes in the blue module. (G) Bubble plot showing BP, CC and MF terms for genes in the blue module. (H) Scatter plot of module eigengenes in the turquoise module. (I) Bubble plot showing BP and CC terms for genes in the turquoise module (MF terms not enriched). WGCNA, weighted gene co-expression network analysis. TOM, topological overlap matrix. BP, Biological Process. CC, Cellular Component. MF, Molecular Function. [file 13578_2023_1034_MOESM8_ESM.tif]

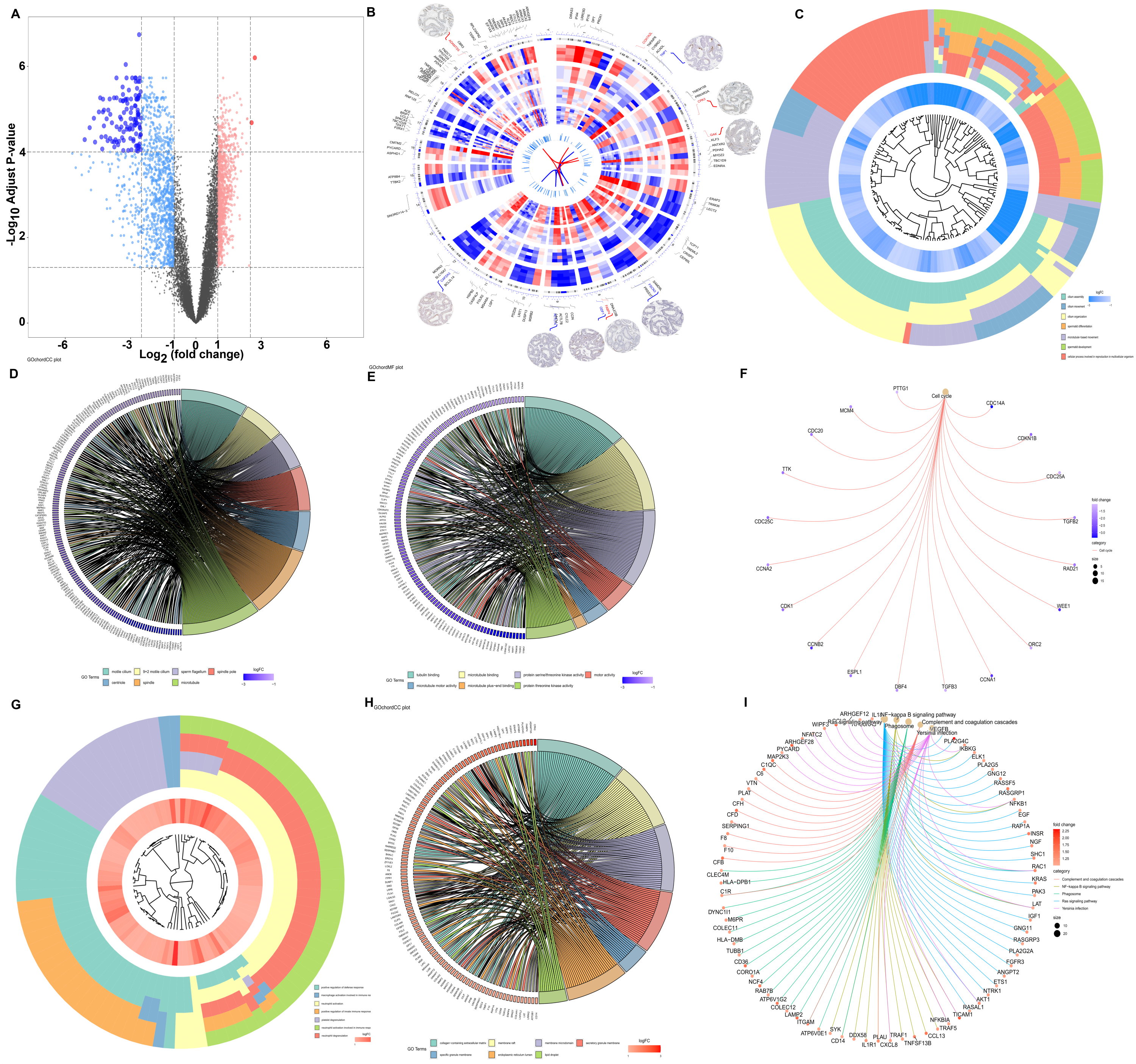

Supplement: Supplementary file 9 — Additional file 9: Figure S7. Identification and functional annotations of DEGs in the discovery set. (A) Volcano plot showing DEGs of the discovery set. Spots in black represented not significantly differentially expressed. Spots in red were upregulated genes with “log2 fold change > 1.0 and adjusted p < 0.05” (light red) or “log2 fold change > 2.5 and adjusted p < 0.0001” (dark red). Spots in blue were downregulated genes “log2 fold change < -1.0 and adjusted p < 0.05” (light blue) or “log2 fold change < -2.5 and adjusted p < 0.0001” (dark blue). (B) Circos plot of chromosomal positions and expression profile of top 100 DEGs. The outer circle showed chromosomes, and each gene symbol pointed to its specific chromosomal location with a line. Samples from the discovery set were visualized in the inner circular heatmaps. Samples with decreasing Johnsen scores were represented from the inside (JS = 10) to the outside circles (JS = 2). The blue bar charts in the inner layer reflected -log10(adjust p) of each DEG. Red color represented up-regulation while blue showed downregulation. The top 5 up and down-regulated genes according to |log2 fold change| (showed in red and blue, respectively) are linked with red or blue lines in core of the plot and their IHC stained sections in the testis from HPA dataset (except for COX7A2L) were shown close to gene symbols. Note: only 97 DEGs remained after matching the 100 DEGs with the reference list. (C), (D), (E) and (F) GO-BP analysis (circular dendrogram), GO-CC, GO-MF analysis (two chord plots) and KEGG pathways (gene-concept network plot), respectively, in downregulated DEGs. (G), (H) and (I) GO-BP analysis (circular dendrogram), GO-CC analysis (chord plot) and KEGG pathways (gene-concept network plot), respectively, in upregulated DEGs. Note: MF of GO analysis was not enriched in upregulated DEGs. DEGs, differentially expressed genes. GO, gene ontology. KEGG, kyoto encyclopedia of genes and genomes. [file 13578_2023_1034_MOESM9_ESM.tif]

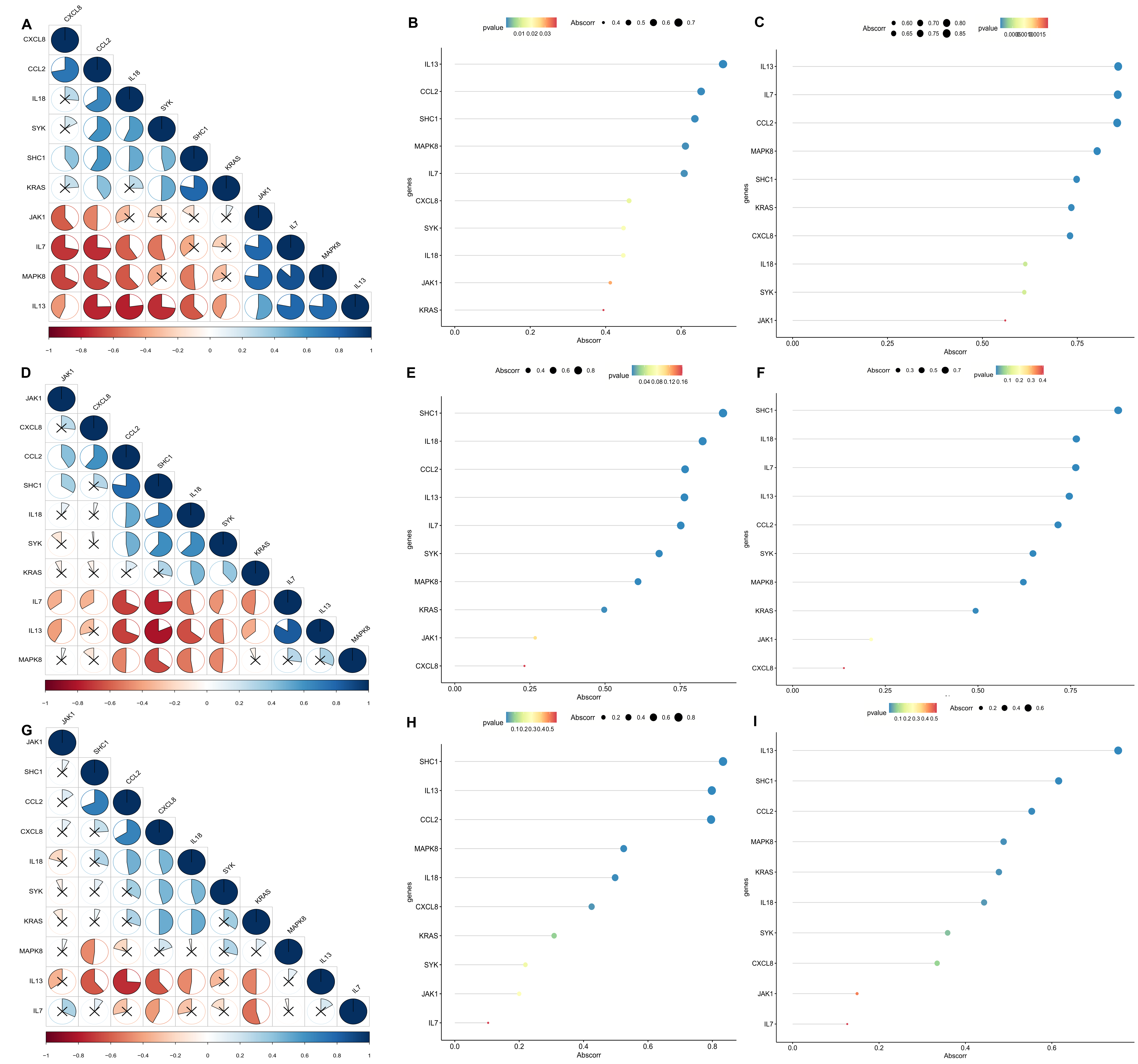

Supplement: Supplementary file 10 — Additional file 10: Figure S8. Internal (discovery set) and external (validation set 1&2) validation of 10 hub immune genes. (A), (D) and (G) Correlation heatmaps of 10 hub immune genes in the discovery set, validation set 1 and validation set 2, respectively. Pie charts showed the proportion of |spearman correlation coefficient| in 1. Blue represented positive correlation while red indicated negative correlation. Color intensity enhanced with the correlation coefficient increasing. A cross on pie chart meant the correlation of the two parameters was not significant (p > 0.05). (B), (E) and (H) Lollipop charts showing spearman correlations of 10 hub immune genes with mast cell infiltration level in the discovery set, validation set 1 and validation set 2, respectively. (C), (F) and (I) Lollipop charts showing spearman correlations of 10 hub immune genes with Johnsen scores in the discovery set, validation set 1 and validation set 2, respectively. Note: To be rigorous, for validation set 2, only samples from spermatogenic dysfunction group with clear JS marked in the original research were analyzed in Figure S8I. For lollipop charts, the length of sticks and the diameter of spots represented absolute value of correlation coefficient (Abscorr). The color of spots varied with p values of correlation analysis. [file 13578_2023_1034_MOESM10_ESM.tif]

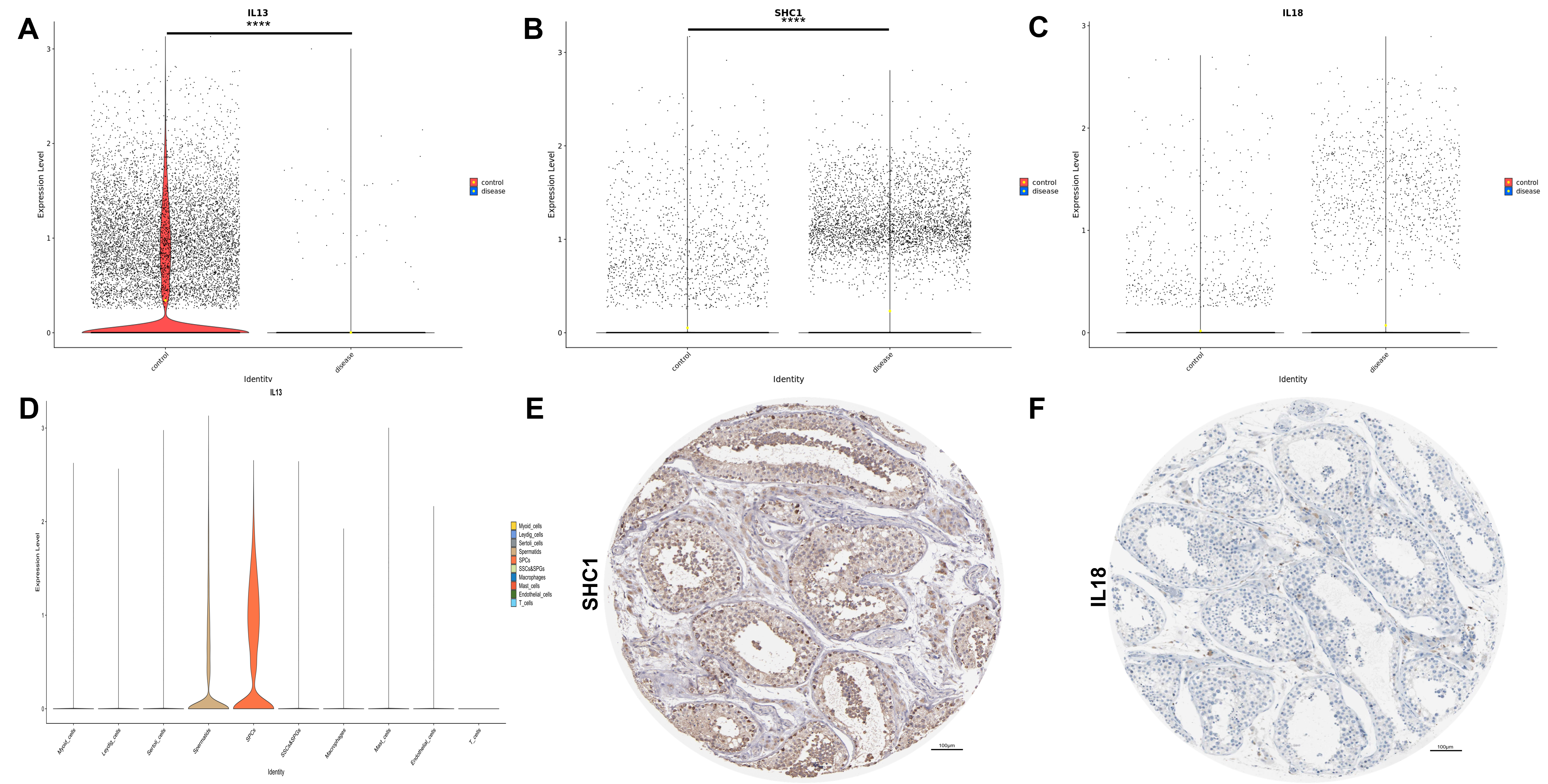

Supplement: Supplementary file 11 — Additional file 11: Figure S9. Expression patterns of selected hub immune genes that show good performances in both internal and external validations. (A)-(C) Violin plot of IL13, SHC1 and IL18 (respectively) expressed in control versus disease groups. Large yellow dots indicated mean expression value, **** adjust p < 0.0001 wilcoxon rank sum test with bonferroni correction using Findmarkers function. (D) Violin plot of IL13 expressed in different testicular cell types. (E) and (F) Immunohistochemical staining of SHC1 and IL18, respectively, in the testis. The original images of IHC stained sections were obtained from Human Protein Atlas database (https://www.proteinatlas.org/). Note that there’s no IL13 IHC staining data of the testis in HPA. [file 13578_2023_1034_MOESM11_ESM.tif]

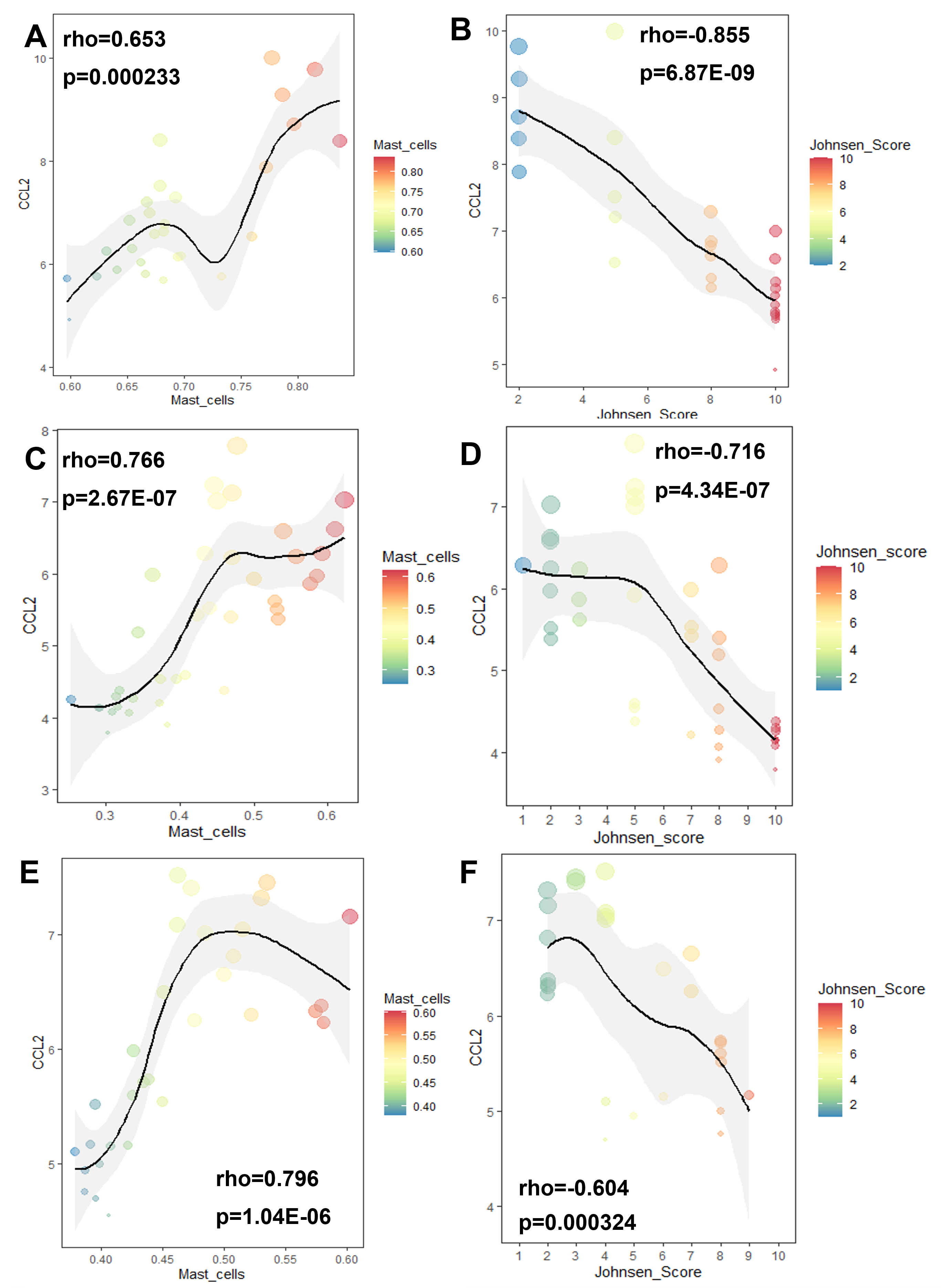

Supplement: Supplementary file 12 — Additional file 12: Figure S10. Scatter plots with loess fitting curves showing the relationship between CCL2 expression and mast cell infiltration (left column) or JS (right column). (A), (C) and (E) Scatter plots showing the spearman correlations between CCL2 expression level and mast cell infiltration level in the discovery set (A), validation set 1 (C) and validation set 2 (E). (B), (D) and (F) Scatter plots showing the spearman correlations between CCL2 expression level and Johnsen scores (or modified Johnsen scores) in the discovery set (B), validation set 1 (D) and validation set 2 (F). Note: To be rigorous, for validation set 2, only samples from spermatogenic dysfunction group with clear JS marked in the original research were analyzed in S10F. [file 13578_2023_1034_MOESM12_ESM.tif]

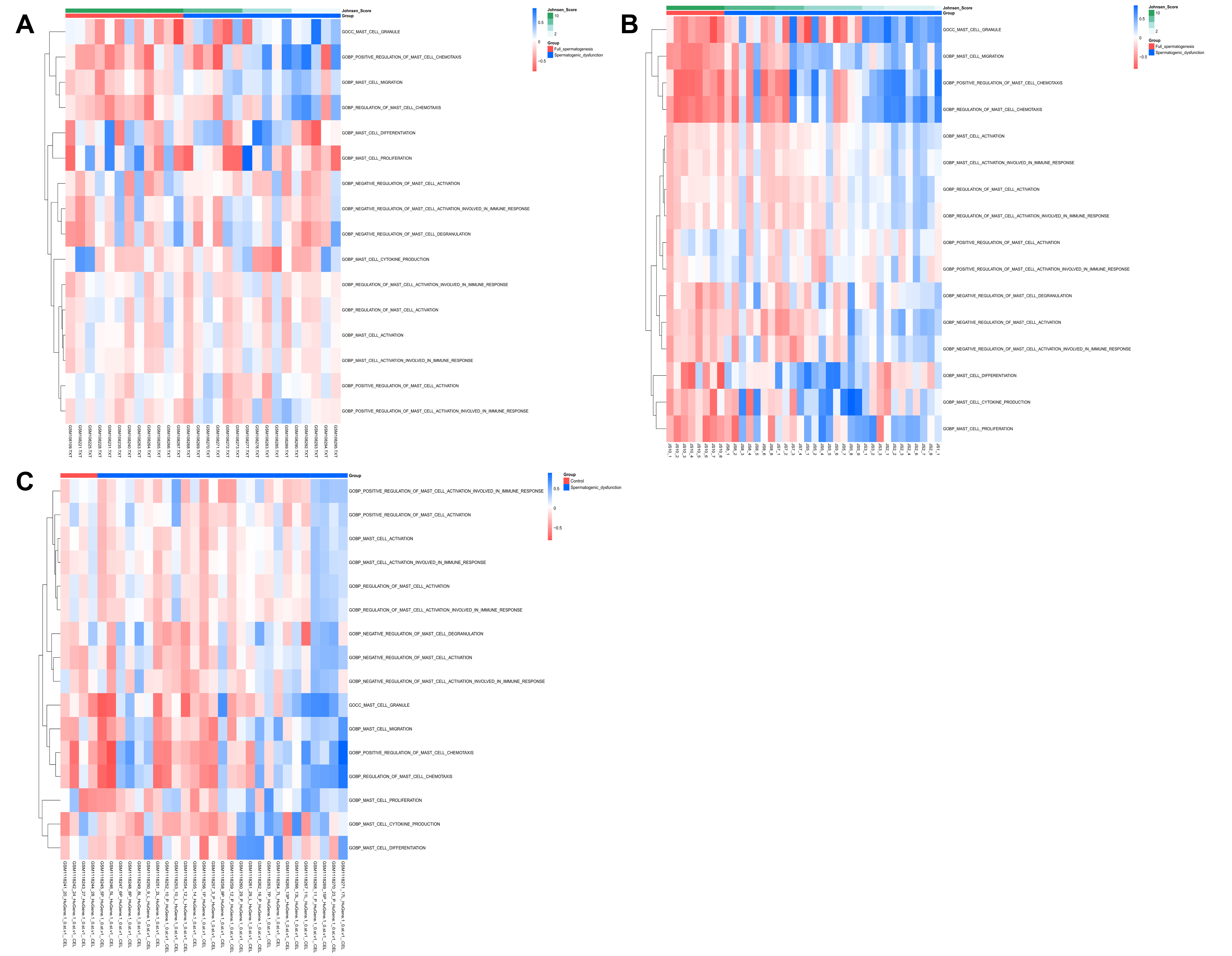

Supplement: Supplementary file 13 — Additional file 13: Figure S11. Heatmaps of scores from GSVA analyses on 16 mast cell related signatures in the discovery set (A), validation set 1 (B) and validation set 2 (C). [file 13578_2023_1034_MOESM13_ESM.tif]

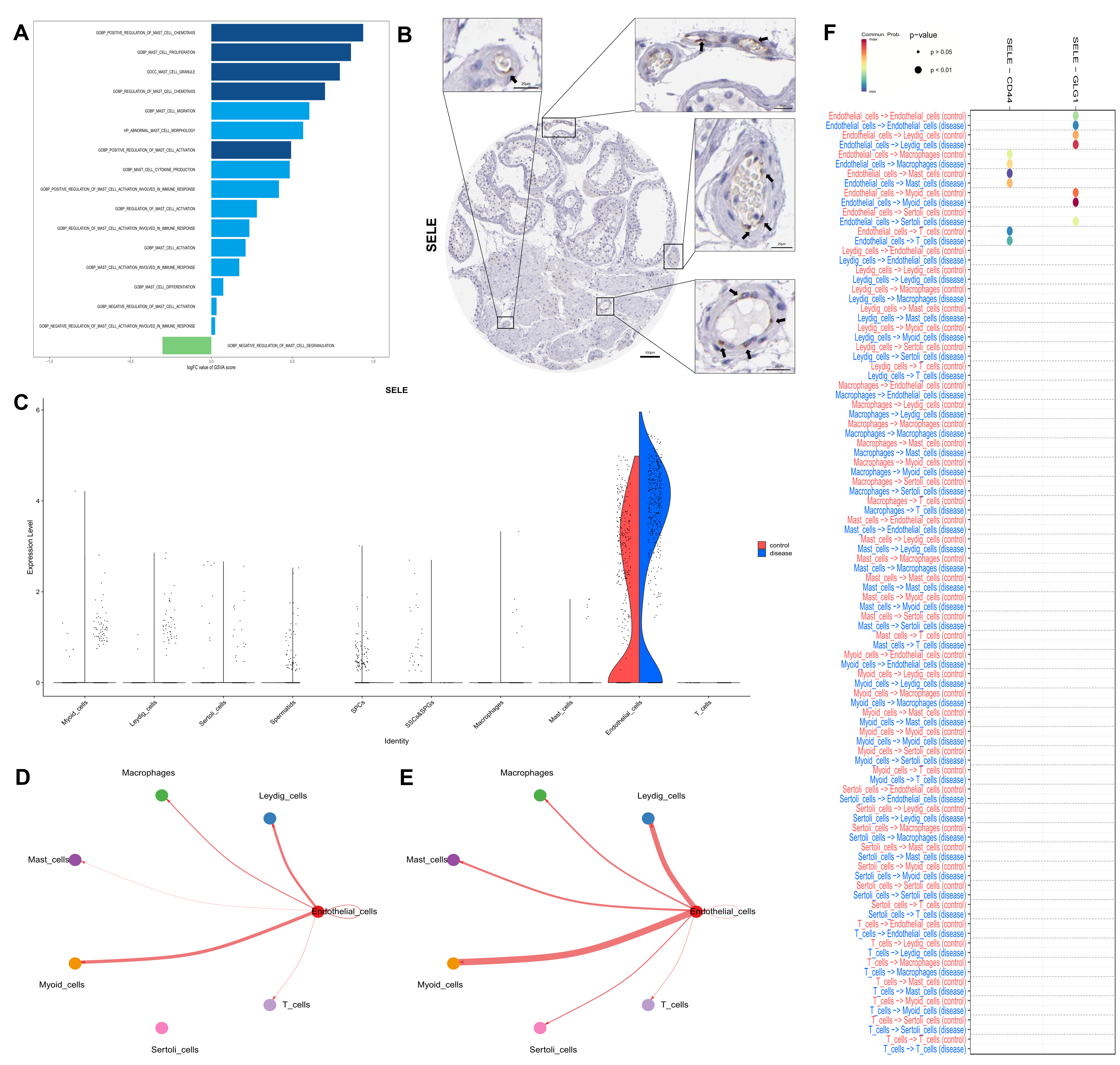

Supplement: Supplementary file 14 — Additional file 14: Figure S12. Potential role of endothelial cells (ECs) in mast cell chemotaxis. (A) Bar plot showing log2 fold change (logFC) of mast-cell related pathways GSVA scores originated from ECs (logFC were obtained by comparing scores from ECs in disease group versus control group). Blue color indicated enhancement in the ECs of disease group. Dark blue meant between-group adjust p < 0.05 (BH adjustment using the limma package). (B) Immunohistochemical staining of SELE in one testis. Arrows represented potential positive cells. The original image of SELE IHC stained section was obtained from Human Protein Atlas database (https://www.proteinatlas.org). (C) Violin plots showing expression level of SELE among different testicular cell types in testes. The violin plots were split by groups. (D)-(E) Circle plots reflecting SELE signal pathway networks in control (D) and disease (E) groups. (F) Bubble plot of detailed SELE signal pathways in cell–cell chats of testes. [file 13578_2023_1034_MOESM14_ESM.tif]
